# Genetic variation shapes the chromatin accessibility landscape and transcriptional responses in mouse adipose tissue

**DOI:** 10.1371/journal.pgen.1011716

**Published:** 2026-01-16

**Authors:** Juho Mononen, Mari Taipale, Marjo Malinen, Anna-Liisa Levonen, Anna-Kaisa Ruotsalainen, Luke Norton, Sami Heikkinen

**Affiliations:** 1 Institute of Biomedicine, University of Eastern Finland, Kuopio, Finland; 2 A.I. Virtanen Institute for Molecular Sciences, University of Eastern Finland, Kuopio, Finland; 3 Department of Environmental and Biological Sciences, University of Eastern Finland, Kuopio, Finland; 4 Department of Forestry, Food Technology, and Environmental Technology, South-Eastern Finland University of Applied Sciences, Kouvola, Finland; 5 Diabetes Division, Department of Medicine, University of Texas Health San Antonio, San Antonio, Texas, United States of America; 6 Department of Cell Systems and Anatomy, University of Texas Health San Antonio, San Antonio, Texas, United States of America; The University of Edinburgh, UNITED KINGDOM OF GREAT BRITAIN AND NORTHERN IRELAND

## Abstract

Most of the disease associated genetic variants identified in genome wide association studies have been mapped to the non-coding regions of the genome. One of the leading mechanisms by which these variants are thought to affect disease susceptibility is by altering transcription factor (TF) binding. Even though inbred mouse strains have been commonly used to investigate polygenic diseases, less is known on how their genetic differences translate to the level of gene regulation and chromatin landscape. Here, we investigated how genetic variation affects chromatin accessibility in the epididymal white adipose tissue (eWAT) of C57BL/6J and 129S1/SvImJ mice, which are commonly used to study diet-induced obesity, fed either chow or high-fat diet. We show that differences in chromatin accessibility are almost exclusively strain-specific and driven by genetic variation. In addition, we integrate ATAC-seq (chromatin accessibility) and H3K27ac ChIP-seq (active regulatory regions) data to show that tissue-specific TF binding sites are commonly found in the active regulatory regions hosting TF motif altering variants in eWAT. Using footprint analysis, we also show that TF occupancy is consistent with TF binding motif scores at the genetically altered loci. In addition, we validate these findings by extending the analysis to ATAC-seq and H3K27ac ChIP-seq data obtained from the liver. We employ RNA-seq to show that differentially expressed genes are co-located with differentially accessible regions hosting genetic variants. Overall, our findings highlight the connection between differential chromatin accessibility and genetic variation across metabolically central tissues of a mouse model for polygenic obesity.

## Author summary

Most of the variants associated with polygenic diseases are outside the protein coding regions of the genome. In this study, we took benefit of the known genetic differences of two inbred mouse strains that are commonly used to study polygenic obesity to investigate the impact of the non-coding genetic variation on the chromatin landscape and the regulation of gene expression in the adipose tissue. Using several complementary sequencing and bioinformatic methods, we show that chromatin accessibility at genetically different transcription factor (TF) binding sites is associated with the strength of the altered TF binding motifs. We also identify the key TFs bound to the regions of genetically determined chromatin accessibility. We further show how the genetic effects propagate to the level of gene expression. Together, these findings give valuable insight into the mechanisms by which genetic variation affects gene regulation and provide future targets for research.

## Introduction

Inbred mouse models have been used to study the mechanisms behind polygenic diseases such as multifactorial obesity [1]. C57BL/6J (B6) is one of the most used models for diet-induced polygenic obesity and is often studied together with mouse strains that are resistant to diet-induced obesity such as 129S1/SvImJ (129). However, most of the research has focused on identifying the key regulatory regions and the underlying biology differentiating the dietary responses of 129 and B6 mice whereas the more subtle effects of genetic variation have not been thoroughly studied [1–3]. As most of the single nucleotide polymorphisms (SNPs) found in genome-wide association studies on, e.g., polygenic obesity are located within the non-coding genome, future research would benefit from understanding how these variants effect the gene regulatory landscape [4]. One of the proposed mechanisms by which these regulatory SNPs (rSNPs) work is that they alter transcription factor (TF) binding [5,6]. Most recently, several studies have demonstrated this phenomenon, but the picture is far from complete [5–7]. As previous studies have shown that several regulatory signals show strain-specific effects between B6 and 129 mice, advancing the understanding on how genetic variation links to the regulatory landscape in different metabolically relevant tissues upon dietary perturbation is paramount.

Previously, differential chromatin accessibility has been shown to be affected by genetics but not diet [2,6]. Assay for Transposase-Accessible Chromatin sequencing (ATAC-seq) has been used widely to assess the effects of genetic variation to regulatory landscape as it can be used to detect candidate TF binding sites and has been shown to correlate well with nearby gene expression [6,8–10]. Distinct classes of TFs relate differently to the regulation of chromatin accessibility. Most notably, pioneer TFs can initiate the opening of closed chromatin regions and thus activate gene expression by allowing other co-operative TFs to bind chromatin [11]. As TFs bind in a sequence-specific manner, genetic variation at the binding sites is known to affect TF binding and, in the case of pioneer factor binding, chromatin accessibility [5,6,12]. Recently, several studies have shown that TFs can have cell-type-specific properties related to chromatin accessibility moderation, *i.e.,* pioneer-like activity, which highlights the need for addressing the complex relationship of TF binding, genetics and chromatin accessibility in more depth [13–18]. In addition, studies on the regulatory landscape in complex diseases have often focused more on the analysis of gene expression data (*i.e.* eQTL analysis) when estimating the effects of genetic variation on the events that contribute to the differentiation of cell types and thus additional studies to identify cell type specific TFs and how their function is affected by genetic variation are needed [19,20].

As shown previously by us and others, differentially accessible regions (DARs) are often enriched for genetic variation and disrupted TF binding motifs [6,8,9,21]. However, looking purely at altered TF binding site motifs in DARs has provided poor accuracy for characterizing rSNPs with roughly ~20% of altered motifs disrupting TF binding [5,6]. For better accuracy, additional methods, such as ChIP-seq, are needed, making the identification of novel interactions cumbersome [6,22]. To study the complex landscape of polygenic diseases where multiple different TFs can be affected, more comprehensive methods for identifying candidate rSNPs must be explored. Footprint analysis is a computational method that identifies TF occupancy sites from accessibility sequencing assays such as ATAC-seq [23]. A recent study by Viestra *et al.* highlighted the usefulness of footprinting in identifying candidate rSNPs by showing that TF footprints were enriched for genetic variation compared to the rest of the non-coding genome [24]. However, footprinting has not been tested in identifying regulatory variants in inbred mice.

In this research we study the relationship of genetic variation and the regulatory landscape in epididymal white adipose tissue (eWAT) of B6 and 129 mice [1]. We show that in eWAT the genetic effects on chromatin accessibility are highly strain-specific and independent of diet-induced effects. We identify active regulatory regions from ATAC-seq and H3K27 acetylation (H3K27ac) ChIP-seq data using an integrative approach which has been previously shown by us and others to increase the accuracy *in silico* TF binding site identification [6,25]. Moreover, we use RNA-seq to show that DARs are located near, and correlate with, differentially expressed genes (DEGs) especially at the promoters. Using footprint analysis of ATAC-seq data, we further show that genetically determined chromatin accessibility co-locates with altered TF binding motifs and that ~75% of the motif scores are altered conjointly with the local accessibility. For additional validation, we expand the footprint analysis to our previously published dataset on the genetically driven regulatory landscape in the liver [6]. These findings also present a compelling case for future studies focusing on genetic variation and TF-driven chromatin accessibility to better characterize the cell type specific pioneer-like activity of TFs the context of regulatory cascades of gene expression.

## Results

### Differences in chromatin accessibility are evident between the strains but not induced by the diet

We used ATAC-seq to identify accessible chromatin regions in the eWAT. This analysis yielded 98,214 nucleosome-free regions (NFRs) across the samples (5–6 samples for 129 and B6 strains on both chow and HFD). Preliminary analysis suggested a possible confounding within the sample set as a set of seven samples clustered without clear strain or diet association (S1A Fig). Sampling eWAT can be difficult due to the heterogenous nature of adipose tissue and the proximity of epididymis [26,27]. To investigate whether the observed confounding could rise from different cell types being present in the samples of the separate cluster (Cluster 2), we investigated the enrichment of the DARs more accessible in Cluster 2 than in Cluster 1 (FDR < 0.01 and fold change > 50%) to cell type-specific genes using a single cell RNA-seq reference from B6 eWAT [28]. This analysis suggested spermatozoa and epididymal cells to be the likeliest source of the observed confounding (S1B Fig). Therefore, we performed our main DAR analysis using csaw by batch-correcting for epididymal/spermatozoa cluster membership which successfully eliminated the bias (S1C-D Fig).

**Fig 1:**
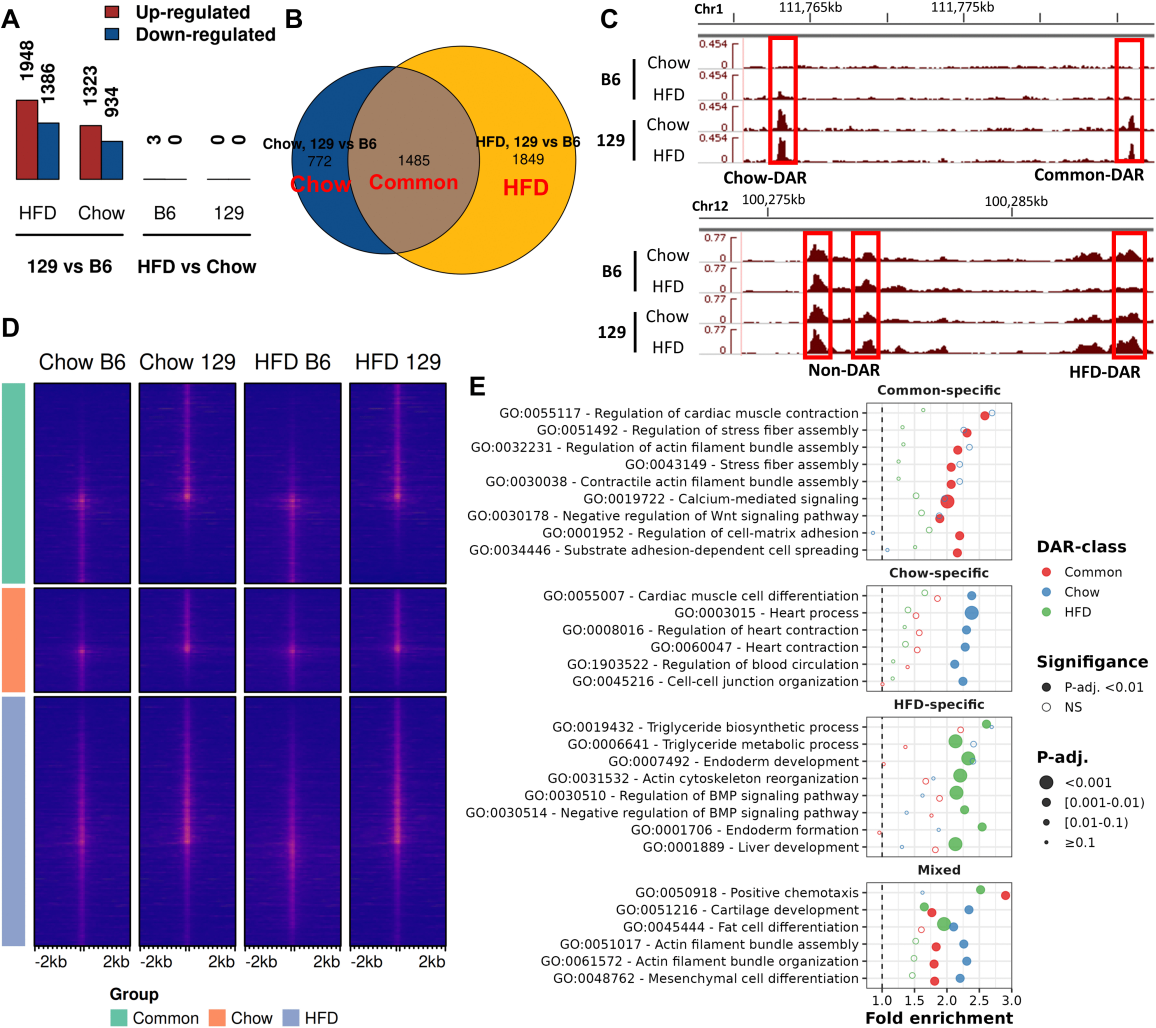
Differences in chromatin accessibility are almost exclusive between the strains and enriched for genetic variation. A) Bar plot of counts of DARs (FDR < 0.01 and fold change > 50%) observed in different comparisons. B) Venn diagram of DAR counts in comparisons with any DARs. DAR-classes^1^ labelled with red. C) ATAC-seq signal tracks highlighting different DARs belonging to different DAR-classes^1^. D) Heatmap of normalized ATAC-seq signal centred on the peak centre across the DAR-classes^1^. E) Results from GREAT analysis of DAR-classes^1^. Top 10 pathways (with hypergeometric test FDR < 0.01) were selected from each DAR-class and results were plotted for all classes. Facets based on the specificity of enrichment for each DAR-class with Mixed being observed in more than one DAR-class. ^1^Common = ”DAR in both diet comparisons”, HFD = ”DAR in HFD comparison”, Chow = ”DAR in chow comparison”.

Regardless, almost no diet-induced changes in chromatin accessibility were observed in either strain, whereas hundreds of inter-strain DARs were detected (Fig 1A). About one third of the 4,105 DARs were common in the two diet comparisons (Fig 1B). Due to the scarcity of diet-induced changes and to keep the analysis focused on the genetics underlying differential accessibility, in subsequent analysis we focused on the inter-strain DARs, classifying each as ‘Chow-DAR’ (DAR only on chow), ‘HFD-DAR’ (DAR only on HFD), or ‘Common-DAR’ (DAR on both diets) (Fig 1B–1D). We also performed enrichment analysis for the DAR-classes to neighbouring genes using GREAT (Fig 1E). Both Chow- and Common-DARs were enriched, either specifically or together, for GO biological processes related to actin filaments, such as “Actin filament bundle assembly”. This highlighted the biological relevance of DARs as actin depolymerization has been characterized as a key step in adipocyte differentiation [29]. On the other hand, the HFD-DARs were specifically enriched to processes of lipid metabolism and Bone Morphogenetic Protein (BMP) signalling, which is known to be connected with adipose tissue function and adipogenesis [30].

### Largest differences in chromatin accessibility are driven by genetic variation

**Fig 2:**
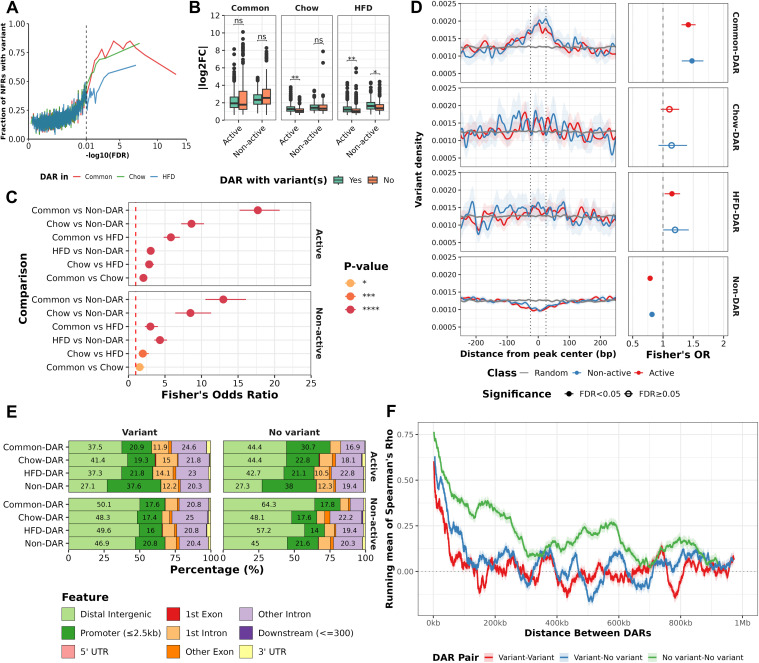
Differential chromatin accessibility locates in distal regulatory regions and spans to nearby DARs. A) The fraction of NFRs containing at least one genetic variant (Y-axis) in windows of 100 NFRs ordered by the significance of difference within diet comparisons (X-axis). For each Common-DAR, FDR is the more statistically significant of the two underlying comparisons, *i.e.,* the test between strains either on Chow or HFD. B) Boxplot of absolute log2 fold changes in different DAR-classes^1^ and Activity classes^2^ comparing between DARs with and without variants (Wilcoxon test^3^). C) Fisher’s test odds ratio^3^ for the enrichment of DARs with variants compared to DARs without variants between different DAR-classes^1,2^. D) Kernel density profile of genetic variants around peak centres of DAR-classes^1,2^ (±250 bp, left panel) and statistical enrichment (Fisher’s test, right panel) of variants in peak centre (±25 bp, dotted lines) and surrounding regions (±26-250 bp) compared to a background of random genomic regions. E) Genomic distribution of NFRs from different classes^1,2^. Percentages >10% labelled. F) Rolling mean of the Spearman’s correlation coefficient (R) (Y-axis) for significantly correlated pairs (P-value < 0.05), plotted against the genomic distance between DARs (X-axis, up to 1 MB). The rolling mean was calculated using a window size of 501 pairs with a step of 100 pairs. The analysis is shown separately for DAR pairs with or without variants. ^1^Common = ”DAR in both diet comparisons”, HFD = ”DAR in HFD comparison”, Chow = ”DAR in chow comparison”, Non-DAR = “Non-DAR in both comparisons”. ^2^Activity-class: Active = NFR flanked by H3K27ac signal, Non-active = NFR not flanked by H3K27ac signal. ^3^P-value: ns ≥ 0.05, * < 0.05, ** < 0.01, *** < 0.001, **** < 0.0001.

Differential accessibility displayed high concordance with genetic variation as higher significance DARs were more likely to overlap genetic variants (Fig 2A). To assess the potential gene regulatory impacts of genetic variation, we categorized those NFRs as Active that were surrounded by the H3K27ac signal, and the rest as Non-active (S2 Fig) [6]. Most of the NFRs, 64–80%, in each DAR-class were observed to be flanked by H3K27ac signal (S3A Fig). DARs observed only on one diet had somewhat different characteristics than the Common-DARs. Both Active and non-active Common-DARs had higher absolute fold-changes than the Chow- or HFD-DARs (Wilcoxon test P-value < 0.001, Fig 2B). In addition, Active Chow- and HFD-DARs displaying higher fold-changes than non-active in the presence of variants while similar effect was not observed with Common-DARs (Fig 2B). While all DARs were enriched for genetic variants compared to non-DARs, Common-DARs also displayed enrichment over both Chow- and HFD-DARs (Fig 2C). This difference was especially clear for HFD DARs, 38.2% of which overlapped with genetic variants, compared to the 78.3% of Common-DARs (S3B Fig). In addition, Active and Non-active DARs had similar proportions of overlapping SNPs with both Common- and Chow-DARs but Non-active HFD-DARs were more enriched for genetic variation compared to Active HFD-DARs (S3B Fig). Furthermore, we observed a higher density of SNPs in the peak centres of Common-DARs compared to both random genomic regions and non-DARs (Fig 2D). While significant enrichment for variants in the peak centres of HFD-DARs was observed the effect size was minimal compared to Common-DARs. In addition, non-DARs were seemingly depleted for genetic variation (Fig 2D).

### Differential accessibility is prominent at distal regulatory regions

Next, we wanted to investigate how the differential accessibility relates to the chromatin landscape. In all DAR classes, including Non-DARs (not DAR in any comparison), and at all types of genomic features, active NFRs were more accessible than non-active NFRs (S3C-D Fig). Active DARs with and without variants were enriched to distal regulatory regions compared to Non-DARs and depleted from the promoter regions (Figs 2E and S3E), even if in general the chromatin was most accessible at the promoter regions of expressed genes (S3D Fig). To investigate if genetically determined chromatin accessibility propagates to nearby regions, we linked DARs located within 1MB of each other and performed Spearman correlation analysis using normalized counts for DARs in all samples. DARs often correlated positively (P-value < 0.05 and Spearman’s Rho > 0) with their nearest DARs especially within approximately 100kb window (Fig 2F). However, DARs with variants were less correlated with their nearby DARs with variants compared to DARs without variants, and positive correlation was most frequent, and occurred over greater distances, between DARs that both are without variants (Figs 2F and S3F). This could suggest that genetic variation generally disrupts the local chromatin landscape instead of propagating to all nearby NFRs.

### Differential gene expression is more pronounced between the strains than diets

**Fig 3:**
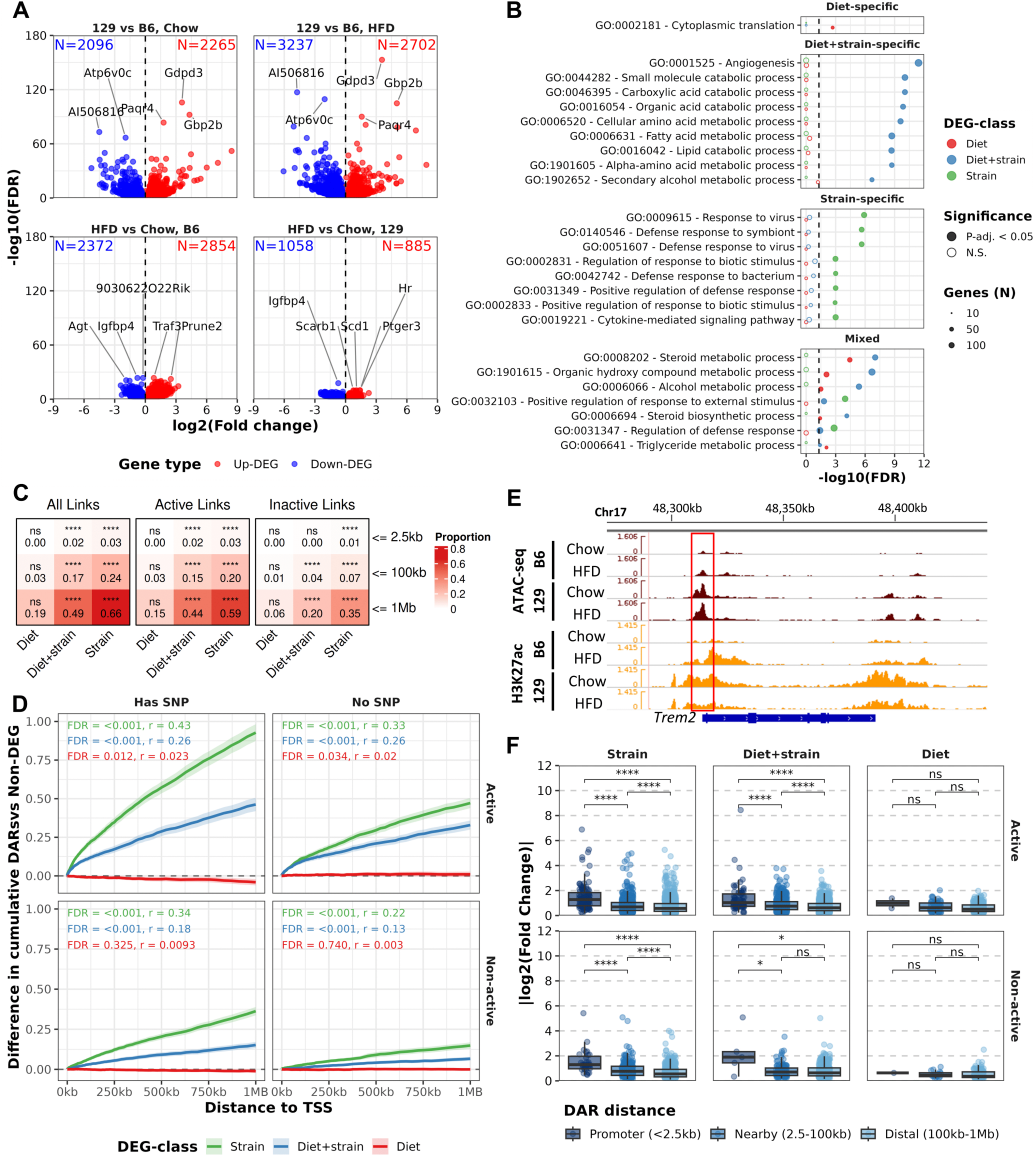
Strain-specific gene expression is connected with genetically determined chromatin accessibility. A) Volcano plots of differentially expressed genes by comparisons. Top 5 most significant (FDR < 0.05) DEGs labelled. B) GO-term over representation analysis results for DEG-classes using expressed genes as the background. Top 10 pathways (with FDR < 0.05) were selected from each DEG-class^1^. Facets based on the specificity of enrichment for each DEG-class with Mixed being observed in more than one DEG-class. C) Heatmap displaying fractions of DEGs^1^ with correlating (Spearman’s Rho > 0 and P-value < 0.05) DARs in cumulative windows from the TSS of the DEG. Fractions and results from statistical testing (Fisher’s test^2^) comparing to non-DARs labelled. Panels from left to right for all DARs and DARs belonging to different activity-classes^3^. D) Difference in cumulative DARs in 1MB window from TSS for DEG-classes^1^ compared to Non-DEGs. Panels separated based on Activity class^3^ and DAR overlap with genetic variants. Statistical testing with Wilcoxon test annotated as text (r = Wilcoxon effect size) E) ATAC-seq and H3K27ac signal tracks in the Trem2 locus. F) Absolute log2 fold-changes of DEGs^1^ with nearest DAR (of any class) in different windows around active TSS. Statistical testing with Wilcoxon test^2^. ^1^Non-DEG = non-DEG in all comparisons, Diet = DEG only in diet comparison, Diet+strain = DEG in both diet and strain comparisons, Strain DEGs = DEG in both strain comparisons. ^2^P-value: ns ≥ 0.05, * < 0.05, ** < 0.01, *** < 0.001, **** < 0.0001. ^3^Activity-class: Active = NFR flanked by H3K27ac signal, Non-active = NFR not flanked by H3K27ac signal.

To enable linking the differences in chromatin accessibility to gene expression, we generated eWAT RNA-seq data for the same mice as for ATAC-seq. Similar to ATAC-seq, preliminary analysis of cluster-wise differentially expressed genes (DEGs, FDR < 0.05, S1 Table) suggested confounding of epididymis origin (S4A and S4B Fig). After successful batch correction (S4A and S4C Fig), the differences between the strains were again the most numerous and with higher effect sizes compared to differences between diets (Fig 3A). Among the most significant DEGs between strains were *Paqr4* and *Gbp2b* (*Gbp1*), both of which have well documented roles in adipocyte function and macrophage polarization, respectively [31,32]. To test whether the cell-type-based batch correction method we used led to drastically altered cell type compositions as inferred from RNA-seq, we performed single cell RNA-seq based deconvolution using BisqueRNA and publicly available data from eWAT of B6 mice [28,33]. The estimated cell type compositions before and after batch correction did not differ within the different groups (Wilcoxon ranked-sum test P-value > 0.05, S4D Fig) and most of the groups presented similar cell type proportions compared to the single-cell reference (S4E Fig). For downstream analyses, we categorized genes to four classes; Non-DEG (not DEG in any comparison), Diet-DEG (DEG only in at least one diet comparison), Diet+strain-DEG (DEG in at least one diet and one strain comparison) and Strain-DEG (DEG in both strain comparisons). We observed that strain-DEGs were most enriched to immune system related pathways whereas Diet+Strain DEGs and Diet DEGs were mostly enriched to pathways related to metabolism (Fig 3B).

### Differentially expressed genes have multiple correlating accessible regions in their neighbourhoods

Next, we wanted to identify how chromatin accessibility relates to gene expression by linking DARs and DEGs by correlating normalized count data from both ATAC- and RNA-seq (Spearman’s correlation, P-value < 0.05, Rho > 0). Majority of the Strain-DEGs had an Active-DAR and/or DAR with variant within 1MB. Both Strain- and Diet+strain DEGs showed significant enrichment of DARs that correlated with their expression (Spearman’s rho > 0, P-value < 0.05) in their neighbourhood compared to Non-DEGs (Fig 3C). This enrichment was observed with both Active and Non-active DARs as well as DARs with or without variants (Figs 3C and S5A). In addition, there were generally more DARs, especially Active DARs overlapping with variants, in the neighbourhoods of DEGs presenting strain-specific expression (Fig 3D). While DARs at promoter were rare, they also displayed stronger correlation on average compared to distal DARs linked to their nearest DEG (S5B and S5C Figs). This was especially clear for both Strain- and Diet+strain-DEGs, where ~80% of DARs correlated positively with DEGs (S5C Fig). For example, DAR overlapping a variant in the promoter of Strain-DEG *Trem2* that showed significant correlation (R = 0.89, P-value < 0.001, Fig 3E). *Trem2* is a key regulator in lipid homeostasis affecting metabolic pathways mediated by lipid-associated macrophages in the adipose tissue [34].

We also wanted to address how NFRs are generally found near DEGs regardless of correlation. Linking genes to their nearest NFR provided only minimal differences between the groups (S5D Fig). Strain- and Diet+strain-DEGs with variant-overlapping DAR at their promoter displayed more extreme fold changes than Diet-DEGs (Fig 3F). However, DARs with and without variants did not differ in this regard. Since the distance to the nearest NFRs tended to be longer for genes with lower expression, the incomplete overlap of NFRs and promoters of expressed genes is likely to be explained by the suboptimal sensitivities of both ATAC-seq and H3K27ac ChIP-seq at the promoters of low expressed genes (S6A and S6B Figs). These findings confirm a link between genetically determined chromatin accessibility and gene expression and suggest that regulatory actions from outside the proximal promoter are a major contributor to gene expression.

### Motif analysis connects chromatin accessibility to cell type specific transcription factors

**Fig 4:**
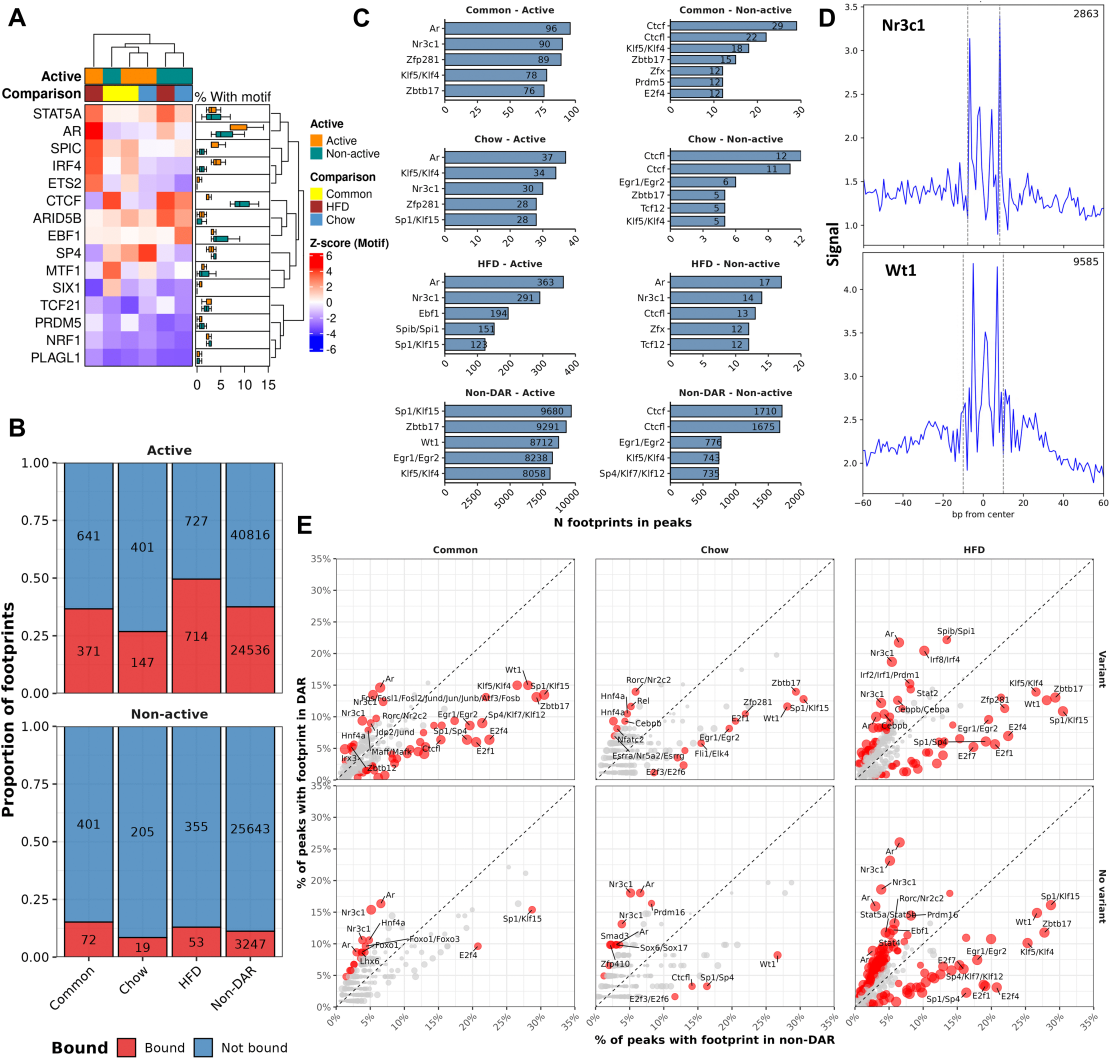
Accessible chromatin overlaps with known adipose tissue transcription factor footprints. A) Heatmap of differential motif enrichment in DAR-classes^1^. Boxplot on the right summarizes the percentages of active and non-active NFRs with motifs across the DAR-classes. B) Fractions of NFRs^1^ that overlap with at least one footprint in eWAT. C) Counts for top 5 most common footprints in active and non-active NFRs^1^ in eWAT. D) Footprint aggregates for NR3C1 (GR) and WT1 across all detected footprints in all groups in the NFRs^1^. E) Scatter plot of footprint occupancy in DARs^1^ vs non-DAR. Size of points is determined by Fisher’s test P-value; red, P-value < 0.05. Top 10 TFs with highest difference in occupancy fraction between DAR-class and non-DAR are labelled (P-value < 0.05). ^1^Common = ”DAR in both diet comparisons”, HFD = ”DAR in HFD comparison”, Chow = ”DAR in chow comparison”, Non-DAR = “Non-DAR in all comparisons”.

After confirming a link between genetically determined chromatin accessibility and gene expression, we looked for key TFs affecting the gene regulatory landscape. Active NFRs were enriched for motifs of known WAT-associated TFs like CEBPs, AR and NR3C1 (also called GR), whereas non-active NFRs displayed high enrichment of the CTCF binding motif, highlighting their potential roles in establishing the chromatin conformation (S7A Fig) [16,35]. Among DARs, Non-active DARs of all classes were similarly more enriched for CTCF binding motifs than active DARs (Fig 4A). In contrast, binding motifs for SPIC, IRF4 and ETS2 were enriched in active Common- and HFD-DARs over all non-active and Chow classes. However, both non-active and active HFD-DARs were enriched for AR and STAT5A binding motifs over other classes. Suspecting that the differences in cell type composition might contribute to the differences between the DAR classes, we performed a chipenrich analysis of DARs and cell type specific genes obtained from the single cell reference we used to assess batch effects [28]. Indeed, the HFD-DARs were the only class of DARs to be enriched to cell type specific genes, namely adipocytes and immune cells (S7B Fig). Combined with the observation that the HFD-DARs had genetic variants less often than the other two DAR classes (Figs 2A and S3B), this suggests that the HFD-specific set of DARs is enriched for cell type specific sites that were detected at least partly due to the increased relative amount of adipocytes and immune cells in the obese B6 eWAT. In summary, DARs with variants might be driving the accessibility of DARs without variants, but accurate interpretation of these observations is hindered by the presence of cell type specific DARs.

### Footprint analysis reveals binding sites for tissue-relevant factors

To move beyond general enrichment of TF binding motifs and to identify candidate TF binding sites more reliably at NFRs, we performed TF footprint analysis using TOBIAS [23]. Footprints identified in the different groups were largely overlapping (S8A Fig). The largest individual groups of footprints were observed with the two HFD groups, which is not surprising since HFD DARs were the largest DAR group. As expected, active NFRs of all classes hosted footprints more frequently than non-active NFRs (Fisher’s test P-value < 0.05, Fig 4B), further highlighting the benefit of separating NFRs based on the flanking H3K27ac signal. Additionally, among the most common footprints in non-active NFRs were footprints for CTCF and its’ paralog CTCFL (Fig 4C). In contrast, active NFRs hosted footprints for several well-known TFs relevant in WAT such as GR (lipolysis and immune response), AR (adipocyte proliferation and metabolism) and WT1 (lipid metabolism) (Fig 4C and 4D) [35,36]. In addition, AR was also present in many non-active HFD-DARs. We also tested whether a given footprint was more common in active DARs compared to active non-DARs. We limited this analysis to those NFRs that had any footprints because the footprint scores, and thus binding classification, depends heavily on the ATAC signal at the NFR, and because substantial differences between the NFR classes were observed (S8B Fig). Quite strikingly, across all DAR sets the results highlighted more frequent binding of AR and GR in DARs compared to non-DARs (Fig 4E). Notably, GR has been shown to represent some pioneer-like activity and has been known to partake in the regulation of AR activity in human adipocytes [35]. However, the motifs for both AR and GR are similar which makes their accurate profiling by footprinting difficult (S9A Fig). Similarity of motifs for other conjointly enriched TFs was also observed (S9A Fig). On the other hand, WT1, which was more frequently bound in non-DARs, is an important regulator for the normal function of WAT, disruption of which can lead to severe developmental deficits [36,37]. As expected, higher proportion of the TFs enriched in DARs were either Strain- or Diet+strain-DEGs compared to those enriched in non-DARs (Fig 9B). Interestingly, of the DAR-enriched eWAT TFs, especially those which were enriched in DARs without variants presented themselves as cell type specific markers in a single cell reference more often than the other groups (S9C Fig).

### The footprint patterns of eWAT are replicated in the liver

To expand the analysis to a larger set of TFs and metabolic functions, we replicated the footprint analysis on our recently published liver dataset [6]. Similar to eWAT, also in the liver the footprints largely overlapped between the groups (S10A Fig). As expected, because the Chow-DARs was the largest DAR group, chow groups had more unique footprints than respective HFD groups (S10B Fig). The active NFRs hosted more footprints, the most frequent being the known hepatic regulators such as HNF4a and the ETS TF family members (S10B and S10C Fig) [38,39]. As expected, CTCFL was the most frequent footprint among the non-active NFR-classes. The enrichment analysis concurred with the frequencies, as, e.g., HNF4a was highly overrepresented in Chow-DARs (S10D Fig). Notably, the liver results replicated the enrichment of SP1 and WT1 footprints in non-DARs also seen in eWAT (S10D Fig). However, the motifs for SP1, a known transcriptional regulator in both eWAT and liver, are similar to WT1 (S9A Fig) [40,41]. Similar patterns seen in both tissues further strengthens the view that genetically determined chromatin accessibility is mediated by a certain set of TFs displaying pioneer activity in the tissue.

### Genetic variation is associated with strain-specific footprints in both eWAT and liver

**Fig 5:**
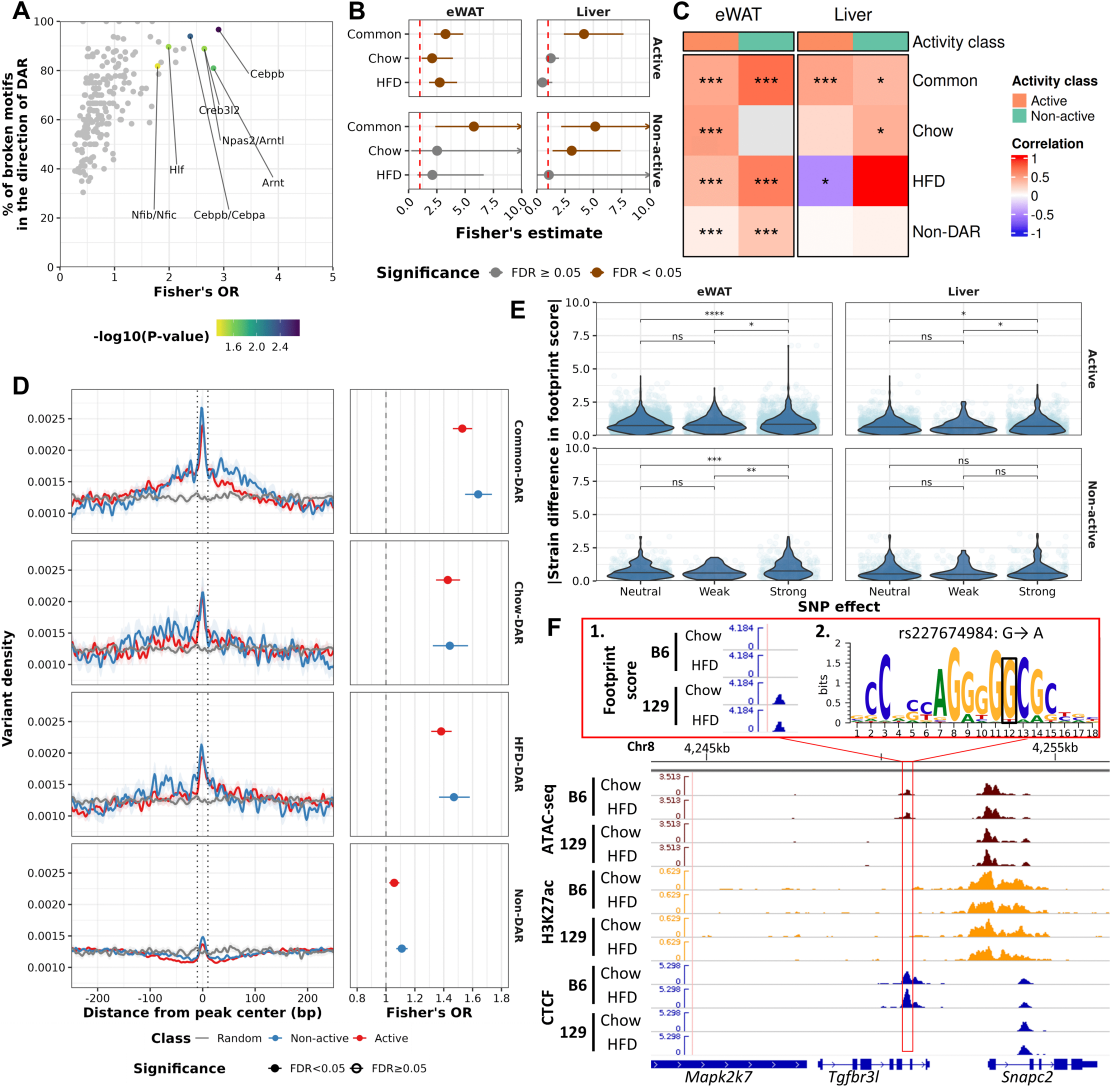
Footprint scores are predictive of motif-altering genetic variation in DARs. A) Comparison of variant-altered *(i.e.,* broken*)* TF binding motifs between Common-DARs and non-DARs using Fisher’s test. TFs with *P*-value < 0.01 are coloured, and further those with >80% of altered motifs with collateral motif score to DAR log_2_FC and *P*-value < 0.01 are labelled. B) Enrichment (Fisher’s test odds ratio) of AC-footprint in DARs compared to non-DARs with non-AC-footprint as background. Upper ends of confidence intervals cropped if upper bound >10. C) Heatmap of Spearman’s correlation coefficients for footprint difference and motif allelic scores within DAR-groups^1^. P-values for correlation coefficients labelled to the heatmap cells. D) Kernel density profile of genetic variants around footprint centres overlapping DAR-classes^1,3^ (±250 bp, left panel) and statistical enrichment (Fisher’s test, right panel) of variants in peak centre (±10 bp, dotted lines) and surrounding regions (±10-250 bp) compared to a background of random genomic regions. E) Violin plots of footprint scores across different strengths of TF binding motif changes caused by genetic variation in eWAT and liver. Statistical testing with Wilcoxon test^2^. F) Genome browser view of the AC-footprint locus for CTCF near *Snpac2* gene overlapping Common-DAR and CTCF DBR in liver highlighted with a red box. Panel 1. Footprint score tracks from TOBIAS. Panel 2. rs227674984 that alters CTCF binding motif in the 129 mice. ^1^Common = ”DAR in both diet comparisons”, HFD = ”DAR in HFD comparison”, Chow = ”DAR in chow comparison”, Non-DAR = “Non-DAR in both comparisons”. ^2^P-value: ns ≥ 0.05, * < 0.05, ** < 0.01, *** < 0.001, **** < 0.0001. ^3^Activity-class: Active = NFR flanked by H3K27ac signal, Non-active = NFR not flanked by H3K27ac signal.

Next, we investigated the impact of genetic variation on the TF occupancy based on strain-specific footprints and chromatin accessibility in general. Similar to our previous findings in liver, altered motifs in the direction of their overlapping Common-DARs were enriched for the motifs of key factors mediating enhancer action, e.g., CEBPB and CEBPA (Fig 5A) [6,42]. There was no significant enrichment for any altered TF motif in the Chow- or HFD-DAR classes. In active NFRs, we identified 1,480 and 573 footprints for eWAT and liver, respectively, where the significantly altered motif allelic score and the corresponding footprint score had the same direction of effect (altered motif corresponding footprint, AC-footprint), (S11A and S11B Fig, S2 Table). DARs, especially Common-DARs in both tissues, displayed significantly higher concordance with AC-footprints compared to non-DARs (Figs 5B, S11A and S11B). Footprint score differences observed between the strains were also positively correlated with allelic motif scores (Spearman’s correlation, P-value < 0.05) and in eWAT this correlation was also observed for Non-DARs (Figs 5C, S11C and S11D). Variants were enriched in the footprint centres for all DAR-classes, with the highest enrichment observed for Common-DARs (Fig 5D). We observed this enrichment also for Non-DARs. In addition, footprints for TFs with corresponding strongly altered motifs (as characterized by motifbreakR) displayed higher footprint score differences compared to neutral and weak motifs in both tissues (Fig 5E). As highlighted before, footprint scores have a strong positive correlation with the ATAC-seq signal and are also affected by cell type specific binding effects, which could influence the detection of genetically determined binding using footprint analysis in non-DARs (S8B and S9C Figs).

Finally, we focused on the identification of transcription factors for which among the altered motifs overlapping with footprints in DARs, to help us understand whether the genetic variation at specific TF binding sites contributes to differential accessibility. The most common AC-footprints in eWAT DARs were those for, e.g., JDP2/JUND and CEBP-family factors which have been suggested, or are known, to have pioneer-like activity (S11E Fig) [43,44]. In the liver the results were quite similar such that the ETS-family and CEBP factors were commonly present among the most frequent AC-footprints (S11F Fig). Unfortunately, due to the low number AC-footprints per TF, inferring the over-representation of TFs in the footprints of DARs provided only minimal results (S12A and S12B Fig). Only one TF in eWAT (STAT6, Common-DAR vs Non-DAR Fisher’s P-value = 0.042) was significantly enriched. However, many of the TFs close to the selected cut-off for significance (P-value<0.05) were mostly concordant by footprint and motif score (S12C and S12D Fig). To assess the reliability of footprints in detecting genetically modified TF binding sites we used our previously published liver CTCF ChIP-seq data [6]. Most of the CTCF footprints overlapped CTCF binding sites (S12E Fig). However, conversely, majority of the CTCF binding sites did not host a footprint (S12E Fig). At differentially bound CTCF sites (DBRs), altered motifs correlated (Spearman’s correlation P-value < 0.05) with footprint scores while at non-DBRs and footprints that were not-overlapping CTCF binding sites similar correlation was not observed (S12F Fig). One interesting locus is near *Snapc2* where a CTCF AC-footprint overlaps both an inactive DAR and a CTCF DBR, with additional nearby Strain-DEGs *Ccl25* and *Prr36* (Fig 5F). While *Snapc2* and *Ccl25* have been associated with liver disease, the function of *Ppr36* has not been fully eluded even though it’s locus hosts variants associated with lipid metabolism by GWAS [45–47]. To summarize, the altered motifs for TFs known to exhibit pioneer activity can be found in the DARs and non-DARs. In addition, the presence of motif-altering variants at footprints is predictive of differential binding at DARs.

## Discussion

In this study we expand on our previous analysis in liver and show how differential chromatin accessibility links to genetic variation and gene expression also in eWAT using a combination of ATAC-seq, RNA-seq and H3K27ac ChIP-seq data [6]. We also inspect how *in silico* inferred binding sites from TF footprinting relate to strain-specific chromatin accessibility.

Similar to our findings in liver, differential accessibility between 129 and B6 mice on chow and HFD was only observed between the strains in eWAT. Regions displaying differential accessibility, especially the most significant ones, were also co-localized with genetic variation. However, unlike in liver, in eWAT we did see pronounced effects of cell type composition affecting both ATAC-seq and RNA-seq samples. Adipose tissue samples have been shown to have highly variable cell type proportions due to tissue heterogeneity, adjusting for which generates more accurate downstream results [26]. Indeed, we identified a subset of samples with significant epididymis-related cell enrichment and employed batch correction for more accurate downstream analysis. Due to the lower number of H3K27ac samples we did not incorporate batch correction there. However, regardless of the batch correction, some signs of differences in cell type composition seem to remain, e.g., because AR and GR footprints were somewhat surprisingly observed also in non-active HFD-DARs, suggesting that the effects of cell type composition propagate also to the H3K27ac signal. Based on our analysis the batch correction did not produce any drastic effects on the cell type composition or artefacts to the processed count data. However, some subtle effects on DAR analysis on the diet comparisons cannot be fully ruled out.

While a strong correlation between DARs without variant and their nearest DAR with variant was observed, DARs without variants presented stronger correlation with other DARs without variants. Given that DARs without variants were most prominent in HFD-DARs which were enriched near adipocyte and immune cell specific genes, it is possible that the stronger correlation is due to differences in the cell type composition as B6 mice have a more pronounced adipose tissue expansion on HFD [1]. In addition, although the existence of multi-NFR chromatin accessibility QTLs (caQTL) has been demonstrated, they are rare when compared to genetically determined single NFRs [21]. In the study by Gate et al. which focused only on CD4 + T-cells, and was thus void off any cell type composition effects, co-accessible peaks only comprised 2% of the total observed NFRs [8]. In addition, it could be possible that the lower correlation between DARs with variant and other DARs is explained by the disruption in the local chromatin landscape mediated by altered TF binding, or that the neighbouring variants not overlapping with DAR could affect chromatin accessibility [21]. It should be noted that some of the strain-specific chromatin accessibility that we observe could be due to trans-effects and need additional validation with more genetic perturbation to be confidently classified as such.

DARs of all classes were predominantly distal to DEGs, but largest effects on gene expression were seen when a DAR with variant was localized at the promoter of a gene. Although DARs were numerous in the neighbourhoods of Strain- and Diet+strain-DEGs, outside of the promoter the fraction of correlating DARs quickly dropped, highlighting that with bulk transcriptomics data the distal NFR-to-gene interactions are hard to interpret. In addition, some sensitivity issues were noticed with the promoter accessibility of lower expressed genes, which may have excluded some of their TSS’s from promoter-based analyses. Single nucleus ATAC-seq has been shown to solve some of the aforementioned issues on cell type specific chromatin accessibility when detecting genetically determined chromatin co-accessibility [48]. However, based on previous studies on caQTLs with or without integration to gene expression, the strongest correlating changes have been observed at short distances, which suggests that the analysis of the proximal connections, *i.e.,* cis-effects, between chromatin accessibility and gene expression are fairly reliable [8,9,21,48]. Then again, studies on genetically determined chromatin accessibility may have missed differences in gene expression that are mediated more distally, by, e.g., trans-effects. It has been proposed that differential accessibility is connected to enhancer priming where lineage-determining factors define the chromatin accessibility landscape, but this does not necessarily translate to differences in gene regulatory landscape without specific stimuli [7,8,14].

Interesting observations were made from the enrichment of footprints between different NFR-classes. For example, DARs in both tissues were enriched for TFs that are known to partake in the differentiation of tissue-specific cells. In eWAT, the highest enrichment in DARs (compared to non-DARs) was observed for GR and AR. The binding motifs for these two TFs are similar, making it difficult to distinguish the actual effector [49]. Although AR has been identified to drive a wide variety of metabolic functions in adipose tissue also in female mice, and not only in males [35,50,51], GR is still known to regulate AR expression in WAT, to be an important factor in adipogenesis, and to present pioneer-like activity [35,52]. Combined, these suggest that it is GR rather than AR that regulates accessibility in eWAT. In addition, it is important to note that although GR and AR are both rapidly exchanging TFs which are often thought to leave no footprints, more recent footprinting methods such as TOBIAS have shown that footprints at the binding sites of these factors can be identified [53–56]. In liver, the most strikingly enriched TF was HNF4α which is a key factor in regulating hepatic progenitor cell formation [57]. Notably, neither GR or HNF4α dysfunction has been shown to have severe phenotypic consequences in their respective tissues [52,58]. In contrast, WT1, which was enriched in non-DARs in both tissues, is an important lineage-defining TF [36,59]. In healthy liver, WT1 is expressed only in the fetal stage where it is an important factor for mesenchymal lineage determination [59]. In WAT, WT1 is necessary for maintaining adipose identity and it is an important factor regulating fat cell development, the dysregulation of which leads to severe developmental deficits [36,60]. This observation could suggest that differential accessibility is driven by more dispensable TFs that could affect cell type composition while alteration in the binding sites of TFs that are required for normal development might not present differential accessibility in adult liver due to more severe consequences. However, it is important to note that the expression of WT1 in adult liver is only observed after liver damage and fibrosis which can affect the conclusions made here [61]. Additionally, WT1 sharing a similar motif with another lineage determining factor SP1 could make interpreting these results difficult [40,41]. We observed that several cell type specific TFs among the enriched TF footprints in DARs did not overlap with genetic variation, suggesting that genetic variation could have more evenly distributed effects across cell types, while DARs without variants are more likely to reflect chromatin accessibility that differs due to differences in cell type composition. As TF expression has been shown to be affected by genetic factors and result in differences in cell type differentiation, additional studies on both *cis*- and *trans-*acting variants on cell type specific TF function are needed [19,20]. However, the overall number of enriched TFs was quite low for DARs with variants which limits the reliability of this interpretation. In summary, TF binding sites for tissue specific TFs partaking in both the regulation and cell type differentiation were often found in DARs, highlighting the benefits of incorporating footprint analysis in the detection of accessibility dependent TFs.

Footprint analysis gave us more insight on which TFs bind at the DARs and non-DARs. However, while the enrichment of altered motifs in DARs vs non-DARs was observed to have similar patterns than in our previous work with liver, enrichment of AC-footprints in DARs did not provide much additional insight [6]. Although we saw CEBP-family TF binding motifs enriched in the altered motif enrichment analysis of DARs, AC-footprints did not show similar pattern of enrichment for these factors. One potential explanation for CEBP-family factors overrepresented in DARs in both tissues could stem from their motif being hypermutable [62]. In addition, while peak-centres of non-DARs were observed to be depleted of genetic variation footprint centres within Non-DARs did show significant enrichment for genetic variants compared to genomic background. This has been suggested to be due to regulatory regions being evolutionary primed for variation in TF binding profiles [24]. Majority of the observed footprints overlapping genetic variants had corresponding changes between their motif and footprint scores, but the number of these instances per TF were too low for meaningful TF-specific statistical analysis. Because DARs in general were less accessible than non-DARs and their accessibility correlated strongly with footprint scores, this could be due to the lack of sensitivity when detecting footprints in DARs, resulting in the low number of observations for AC-footprints. In addition, as recently shown by a study focusing on TCF7L2 function in the hepatocytes, TF binding can be very specific even within a cell type [18]. Such highly cell type specific binding, which is still reflected on chromatin accessibility, is less likely to present with strong signals for footprint analysis or even DAR analysis with bulk ATAC-seq where the signals from several different cell types combine, which could be solved using single cell approaches [24].

In summary, differences in cell type composition, both genetically driven and not, seem to affect the identification of genetically determined TF binding from bulk ATAC-seq datasets. Scores for altered motifs overlapping footprints in DARs correspond with changes in footprint scores, which presents a strong case for the mediation by cell type specific TF occupancy on the genetically determined chromatin accessibility.

## Materials and methods

### Ethics statement

Mice were acquired from Jackson Laboratories (Boston, USA) and housed at the Lab Animal Centre of the University of Eastern Finland. The animal experiments were authorized by the national Animal Experiment Board, Finland (ELLA) under the ethical permit ESAVI-2015–002081.

### Mouse experiments

Male C57BL/6J and 129S1/SvImJ mice were fed either a chow (Teklad 2016, Envigo) or HFD (TD.88137, Harlan) from 9 weeks of age for 8 weeks. At 17 weeks of age, mice were euthanized by CO2 for harvesting tissues. The epididymal white adipose tissue pads were excised avoiding non-adipose tissue, and the total weight was recorded. Two hundred mg piece of each pad as well as all the rest were snap-frozen in liquid N2 and stored in -80°C.

### RNA extraction

Total RNA was extracted from mouse eWAT tissue using the miRNeasy Mini Kit and QIAzol Lysis Reagent from QIAGEN, according to the manufacturer’s protocol. The RNA was treated with RNase-Free DNase (QIAGEN) to remove any DNA. The RNA quality was assessed with an Agilent Bioanalyzer and the RNA 6000 Nano kit, with all samples showing a RIN score of 7 or higher.

### Library preparation and RNA-sequencing

RNA-seq libraries were generated using 800 ng of total RNA. Ribosomal RNA was first removed with the NEBNext rRNA Depletion Kit (New England BioLabs). The libraries were then prepared using the NEBNext Ultra II Directional RNA Library Prep Kit for Illumina (New England BioLabs), according to the manufacturer’s guidelines. The yield of the libraries was assessed using the Qubit DNA High Sensitivity assay (Invitrogen), and quality control was conducted with the Agilent Bioanalyzer using the DNA 1000 kit (Agilent). Finally, the indexed libraries were pooled and sequenced on the NextSeq 500 platform (Illumina) with 75 bp single-end reads.

### Nuclei extraction, library preparation and sequencing for ATAC-seq

Approximately 100 mg of eWAT tissue was used to generate libraries by following the Omni-ATAC protocol [63] with slight modifications. Nuclei were extracted using a Dounce homogenizer and 1 × homogenization buffer composed of 320 mM sucrose, 0.1 mM EDTA, 0.1% NP-40, 5 mM CaCl₂, 3 mM Mg(Ac)₂, 10 mM Tris pH 7.8, 1 × protease inhibitors (Roche, cOmplete), and 1 mM β-mercaptoethanol. After homogenization, the samples were filtered through a 100 µm nylon mesh filter, and nuclei were recovered by OptiPrep/iodixanol (Sigma-Aldrich) density gradient centrifugation. For the transposition reaction, 50,000 nuclei were resuspended in a transposition mix containing 25 µl 2 × TD buffer, 2.5 µl transposase, 16.5 µl PBS, 0.5 µl 1% digitonin, 0.5 µl 10% Tween-20, and 5 µl H₂O. Following the transposition reaction, DNA was purified using the DNA Clean & Concentrator-5 Kit (Zymo Research). Ten microliters of the purified transposed DNA were then amplified for 9–11 cycles using 25 µl of 2 × Ultra II Q5 Master Mix (New England BioLabs) and 1 µl each of the amplification primers (1.25 μM final concentration) [10]. To clean up the libraries and remove primer dimers and fragments larger than 1,000 bp, SPRIselect beads (Beckman Coulter) were used. The library yield was quantified with the NEBNext Library Quant Kit for Illumina (New England Biolabs), and quality was assessed using the Agilent Bioanalyzer and High Sensitivity DNA Kit (Agilent). Sequencing was performed using the Illumina NextSeq 500 platform with paired-end 75 bp reads.

### ChIPmentation-based ChIP-seq analysis of H3K27 acetylation

To perform ChIP-Seq on mouse eWAT, approximately 100 mg of frozen tissue was Dounce homogenized and fixed with 20 strokes using the loose pestle A followed by 20 strokes with the tight pestle B in Cell Lysis Buffer (5 mM PIPES pH 8, 85 mM KCl, 1% Igepal, 1 × protease inhibitor) containing 0.5% formaldehyde. The mixture was then rotated for 8 minutes at room temperature. The cross-linking reaction was quenched by adding 150 mM glycine for 5 minutes at room temperature. The cross-linked samples were subsequently washed twice with ice-cold Cell Lysis Buffer supplemented with 1 × protease inhibitors. The pellet containing the cross-linked chromatin was suspended in 300 µl of SDS Lysis Buffer (50 mM Tris-Cl, 0.5% SDS, 10 mM EDTA, 1 × protease inhibitor) and incubated on ice for 1 hour. Chromatin was sheared using a Bioruptor Plus sonicator (Diagenode) for 35 cycles at a high setting (30s ON, 30s OFF per cycle), allowing the sonicator to cool down for 5 minutes after every 10 cycles. To neutralize SDS, Triton-X-100 was added to a final concentration of 1% along with 1 × protease inhibitors. Samples were centrifuged at 13,000 rpm for 20 minutes, and the supernatant containing sheared chromatin was collected and divided into 140 µl aliquots for immunoprecipitation.

ChIP and library preparation followed the ChIPmentation protocol [64] with minor adjustments. For ChIP, 2 µg of Anti-Histone H3 (acetyl K27) antibody (Abcam, #4729) was added to 50 µl Protein G-coupled Dynabeads (Thermo Fisher Scientific) in 1 × PBS with 0.5% bovine serum albumin (BSA) and rotated at 40 rpm for 4 hours at 4°C. For the IgG control, 2 µg of Rabbit IgG (Diagenode, #C15410206) was used in a similar manner. Antibody-coated Dynabeads were washed three times with PBS containing 0.5% BSA and then mixed with 140 µl of chromatin samples in 1.5 ml tubes, followed by overnight rotation at 40 rpm at 4°C. Immunoprecipitated chromatin was washed with low-salt buffer (50 mM Tris-Cl, 150 mM NaCl, 0.1% SDS, 0.1% sodium deoxycholate, 1% Triton X-100, and 1 mM EDTA), high-salt buffer (50 mM Tris-Cl, 500 mM NaCl, 0.1% SDS, 0.1% sodium deoxycholate, 1% Triton X-100, and 1 mM EDTA) and LiCl buffer (10 mM Tris-Cl, 250 mM LiCl, 0.5% IGEPAL CA-630, 0.5% sodium deoxycholate, and 1 mM EDTA), followed by two washes with TE buffer (10 mM Tris-Cl and 1 mM EDTA) and two washes with ice-cold Tris-Cl pH 8.Immunoprecipitated bead-bound chromatin was then resuspended in 30 µl of 2 × TD buffer and 1 µl of transposase (Nextera, Illumina) for tagmentation. After incubation at 37°C for 10 minutes, the samples were washed twice with low-salt buffer. Bead-bound tagmented chromatin was diluted in 23 µl of water and combined with 25 µl of 2 × Ultra II Q5 Master Mix (New England BioLabs, M0544S) and 1 µl of both amplification primers [10]. Library preparation included adapter extension at 72°C for 5 minutes, reverse cross-linking at 95°C for 5 minutes, followed by 11 cycles of PCR amplification (98°C 10s, 63°C 30s, 72°C 3 minutes). After PCR amplification, double-sided purification was performed using SPRIselect beads (Beckman Coulter). The yield of the libraries was quantified using the Qubit DNA High Sensitivity assay (Invitrogen), and quality assessment was conducted using the Agilent Bioanalyzer with the DNA 1000 kit (Agilent). ChIP-seq libraries were sequenced on the HiSeq3000 (Illumina) platform with single-end 75 bp reads.

### RNA-, ATAC- and ChIP-seq preprocessing

Pseudogenome and transcriptome for the 129 mice were produced as described previously by us except for updating the Gencode transcriptome to M25 [6]. Similarly, the sequencing file preprocessing followed the described protocol apart from using STAR version 2.6.1 for aligning the reads [65].

### Statistical analysis and visualization

Statistical analyses were performed with R software (v4.1.0) [66]. Heatmaps were plotted using ComplexHeatmap (v2.13.3) and EnrichedHeatmap (v1.27.2) [67,68]. Venn diagrams were plotted using nVennR (v0.2.3) [69]. Footprint aggregate plots were generated using TOBIAS (v0.14.0) ‘plotAggregates’ function with default settings [23]. Rest of the plots were generated using ggplot2 (v3.5.1) [70].

### Outlier detection and statistical analysis of ATAC- and RNA-seq

RNA-seq and ATAC-seq samples were tested for outliers using rrcov (v1.7-5) function ‘PcaGrid’ with ‘crit.pca.distances = 0.999’ [71]. Optimal k-value for outlier detection was selected with PCAtools (v2.6.0) function ‘parallelPCA’ with ‘threshold = 0.01, max.rank = 24’ parameters [72]. Before outlier detection, for RNA-seq the read counts were normalized with VST normalization (DESeq2, v1.34.0) and for ATAC-seq with local regression fit using csaw (v1.28.0) [73,74]. After outlier removal, UMAP (umap, v0.2.10.0) was used to determine the existence of clusters deviating from the rest of the samples [75]. Differentially accessible regions (DARs) were detected using csaw with cluster as a covariate from NFRs with log_2_(CPM)> –3 counts. To focus on the Tn5 cut-sites, reads were shortened to 1 bp tags at 5′-ends before read counting. For RNA-seq, DEGs were detected using DESeq2. Only genes that had at least one read in one of the samples were included in the analysis. Single cell data set with normalized counts and cell type annotation for mouse eWAT was acquired from the study by Sárvári *et al.* [28]. Single-cell based deconvolution for RNA-seq was performed using BisqueRNA. Cell type specific markers were detected using Seurat (v4.1.0) ‘FindAllMarkers’ command with ‘only.pos=TRUE’ [76]. Enrichment of cluster DEGs to cell type specific genes was done using clusterProfiler’s (4.2.2) enricher function. For DARs chipenrich’s (v2.17.0) ‘chipenrich’ was used for the enrichment analysis with following parameters ‘method = “polyenrich”, genome = “mm10”, locusdef = “nearest_tss”’ [77,78].

### ATAC-seq analysis

Enrichment of DARs to PANTHER GO slim (v17.0) terms belonging to “biological_process” ontology was analysed with rGREAT (v2.1.11) [79,80]. Motif enrichment analysis for the active and non-active NFRs was done using GimmeMotif’s (v0.18.0) ‘gimme motifs’ and for the different classes of DARs with ‘gimme maelstrom’ [81]. Motifs classified as “Direct” from the CIS-BP v2.0 collection and mm10 (B6) genome were used in the analyses [49]. Footprint analysis was done with TOBIAS using the strain-specific genomes [23]. For footprint analysis, the motifs were subset to those of expressed TF genes and merged with universalmotif (v1.12.3) ‘merge_similar’ with default parameters [82]. Before merging, motifs with average information content ≤ 0.75 were discarded to increase the reliability of the merging. Footprints from the 129 data were converted to B6 coordinates to create a consensus set of TF occupancy sites using g2gtools (v0.29) ‘convert’ [83]. Scores for B6 and 129 mice were then calculated strain specifically for these locations. Genetically altered binding sites were identified using motifbreakR (v2.8.0) with ‘filterp = TRUE, threshold = 1e-4, method = ‘ic’, legacy.score = FALSE, show.neutral = TRUE’ and MGPv5 SNPs and InDels [84]. For testing proportional differences between peak classes Fisher’s test implemented in rstatix was used.

### RNA-seq analysis

To select active genes, zFPKM values for genes were calculated using the zFPKM R package (v1.16.0), considering a gene expressed when it had an average zFPKM > -3 and/or FDR < 0.05 in one of the differential gene expression comparisons [85]. Active transcription start sites (TSSs) were detected using proActiv (v1.4.0) and the most active TSS for each gene were used in the downstream analysis as the gene start point [86]. TSSs classified as Major and Minor by proActiv were prioritized in the selection over internal promoters with higher activity score. Over-representation analysis was performed using clusterProfiler’s ‘enrichr’ using PANTHER GO slim terms (v17.0) [79].

### Integrative analysis of ATAC- and RNA-seq

NFRs were linked to the active TSS’s of genes within 1MB using ChIPpeakAnno (v3.28.0) ‘annoPeaks’ with ‘bindingRegion=c(-1e6,1e6), select=”all”’ parameters [87]. Peaks were assigned to genomic features using ChIPseeker (v1.30.3) ‘annotatePeak’ and expressed genes as annotation [88]. Correlation analyses were done using Spearman rank correlation with logCPM normalized counts from edgeR (v3.36.0) [89].

## Supporting information

S1 Fig Batch correction resolves cell type bias from ATAC-seq data. **A)** UMAP plot based on normalized DAR counts. Cluster 2 is highlighted with red circle. **B)** Chipenrich results for Cluster 2 vs Cluster 1 DARs to cell type-specific genes. Significantly enriched/depleted (FDR < 0.01) gene sets labelled**. C)** UMAP plot of limma batch corrected normalized counts. **D) S**catter plots of mean normalized DAR counts in Cluster 1 (x-axis) and Cluster 2 (y-axis) before and after batch correction.
(TIF)

S2 Fig Enrichment heatmap of normalized ATAC-seq and H3K27ac ChIP-seq signal at DAR-classes^1^ across different Activity classes^2^. ^1^Common = ”DAR in both diet comparisons”, HFD = ”DAR in HFD comparison”, Chow = ”DAR in chow comparison”, Non-DAR = “Non-DAR in both comparisons”. ^2^Activity-class: Active = NFR flanked by H3K27ac signal, Non-active = NFR not flanked by H3K27ac signal.
(TIF)

S3 Fig Active NFRs present higher accessibility than non-active NFRs. **A)** Fractions of NFRs in DAR-classes^1^ separated to activity classes^2^ with or without overlapping genetic variant. **B)** Counts of DARs in different classes^1,2^ with percentages of DARs overlapping with variants labelled. Statistical testing for differential proportions of variants in activity classes with Fisher’s test^3^. **C-D)** Boxplots of normalized counts for active and non-active NFRs in **C)** different DAR-classes^1^ and **D)** different genomic features. **E)** Heatmap of Fisher’s test results on the enrichment of DARs to genomic features compared to non-DARs. **F)** Violin plots of significant Spearman’s correlations between two DARs with or without genetic variant overlap at different distance bins. **C, D, F)** Statistical testing with Wilcoxon signed rank test^3^. ^1^Common = ”DAR in both diet comparisons”, HFD = ”DAR in HFD comparison”, Chow = ”DAR in chow comparison”, Non-DAR = “Non-DAR in both comparisons”. ^2^Activity-class: Active = NFR flanked by H3K27ac signal, Non-active = NFR not flanked by H3K27ac signal. ^3^P-value: * < 0.05, ** < 0.01, *** < 0.001, **** < 0.0001.
(TIF)

S4 Fig Batch-corrected gene expression corresponds to chromatin accessibility at the promoter. **A)** UMAP plot of normalized counts before and after batch correction. Cluster 2 highlighted with a red circle. **B)** MA plots of Cluster 2 vs Cluster 1 normalized counts before and after batch correction. **C)** Over representation analysis results of genes up-regulated in Cluster 2 vs Cluster 1 in sets of cell type specific genes. Bars coloured grey are non-significant (FDR > 0.01). **D)** Violin plots of estimated cell type proportions in batch corrected (batched) and non-corrected (non-batched) sample groups. **E)** Violin plots of estimated cell type proportions in batch corrected samples compared to proportions observed in the scRNA-seq reference dataset. **D-E)** Statistical testing with Statistical testing with Wilcoxon test. P-value: * < 0.05, ** < 0.01, *** < 0.001, **** < 0.0001.
(TIF)

S5 Fig Nearby DARs present higher correlation with DEGs. **A)** Heatmap displaying fractions of DEGs^1^ with correlating (Spearman’s Rho > 0 and P-value < 0.05) DARs (of any class) in different windows from the TSS of the DEG. Fractions and results from statistical testing (Fisher’s test^2^) comparing to non-DARs labelled. Panels for DARs belonging to different activity-classes^3^ **B)** Correlation coefficients (Spearman’s Rho, P-value < 0.05) for DEGs and their nearest DARs (of any class) in different distance categories. Vertical facets for DARs belonging to different activity-classes^3^. Statistical testing with Wilcoxon test annotated as text (r = Wilcoxon effect size) ^2^. **C)** Heatmap of fractions of correlating DARs in DAR-classes^3^ for DEGs in DEG-classes^1^ (horizontal panels) separated by Activity class^4^ (vertical panels), variant overlap, and distance bins. Number of linked DARs annotated as numbers in the heatmap cells. **D)** Circulograms for the fractions of closest NFR distance and class for expressed genes^1^. DAR = Chow-/HFD-/Common-DAR. **E)** Absolute log2 fold-changes of DEGs^1^ (horizontal panel) with nearest DAR (of any class) in different windows around active TSS. Separated for Activity class^4^ (vertical panel). Statistical testing with Wilcoxon test^2^. ^1^Non-DEG = non-DEG in all comparisons, Diet = DEG only in diet comparison, Diet+strain = DEG in both diet and strain comparisons, Strain DEGs = DEG in both strain comparisons. ^2^P-value: ns ≥ 0.05, * < 0.05, ** < 0.01, *** < 0.001, **** < 0.0001. ^3^Common = ”DAR in both diet comparisons”, HFD = ”DAR in HFD comparison”, Chow = ”DAR in chow comparison”, Non-DAR = “Non-DAR in both comparisons”. ^4^Activity-class: Active = NFR flanked by H3K27ac signal, Non-active = NFR not flanked by H3K27ac signal.
(TIF)

S6 Fig Genes without nearby NFRs are lowly expressed **A)** Enrichment heatmap of ATAC-seq and H3K27ac ChIP-seq signals ±2kb from the active TSS of expressed genes. **B)** Scatter plot of normalized expression levels of genes^1^ and their distance to nearest NFR. ^1^Non-DEG = non-DEG in all comparisons, Diet = DEG only in diet comparison, Diet+strain = DEG in both diet and strain comparisons, Strain DEGs = DEG in both strain comparisons.
(TIF)

S7 Fig HFD DARs present adipocyte specific enrichment. **A)** Motif enrichment for group-wise active and non-active NFRs. Heatmap columns and rows are clustered by Euclidean distance and columns are split using *K*-means clustering. Values shown are for the most significant motif for a given TF. Motifs for heatmap were selected by group-wise filtering of redundant motifs. **B)** Fisher’s test results for enrichment of Active and non-active DARs* to cell type specific genes. *Common = ”DAR in both diet comparisons”, HFD = ”DAR in HFD comparison”, Chow = ”DAR in chow comparison”.
(TIF)

S8 Fig Footprints in liver show similar patterns as in adipose tissue. **A)** Venn diagram of footprints observed in the different groups in eWAT. Numeric IDs for overlapping groups are presented in the brackets. 1 = Chow B6, 2 = HFD B6, 3 = Chow 129 and 4 = HFD 129. **B)** Scatter plot of footprint scores and normalized (log2 CPM) ATAC-seq counts in the different NFRs^1^. Correlation testing with Spearman’s correlation. ^1^Common = ”DAR in both diet comparisons”, HFD = ”DAR in HFD comparison”, Chow = ”DAR in chow comparison”, Non-DAR = “Non-DAR in both comparisons”.
(TIF)

S9 Fig Motifs enriched in DARs or non-DARs share similarity. **A)** Motifs clustered by Pearson correlation. Motifs enriched in DARs in red and non-DARs in cyan. **B)** Percentages of enriched TFs in DEG-classes^1^. Counts and percentages labelled. **C)** Proportions of enriched TFs that are positive cell type markers derived from a single cell reference (Adjusted P-value < 0.01 and average log2 fold change > 0.25). Counts and percentages labelled. ^1^Non-DEG = non-DEG in all comparisons, Diet = DEG only in diet comparison, Diet+strain = DEG in both diet and strain comparisons, Strain DEGs = DEG in both strain comparisons.
(TIF)

S10 Fig Footprints in liver show similar patterns as in adipose tissue. **A)** Venn diagram of footprints observed in the different NFRs^1^ in liver. Numeric IDs for overlapping groups are presented in the brackets. 1 = Chow B6, 2 = HFD B6, 3 = Chow 129 and 4 = HFD 129. **B)** Fractions of active and non-active NFRs by NFR-class^1^ that overlap with at least one footprint in liver. **C)** Counts for top 5 most common footprints in active and non-active NFRs^1^ in liver. **D)** Scatter plot of footprint occupancy in DARs vs non-DARs. Size of points is determined by Fisher’s test P-value; red: P-value < 0.05. Top 10 TFs with highest difference in occupancy fraction between DAR-class^1^ and non-DAR are labelled (P-value < 0.05). ^1^Common = ”DAR in both diet comparisons”, HFD = ”DAR in HFD comparison”, Chow = ”DAR in chow comparison”, Non-DAR = “Non-DAR in both comparisons”.
(TIF)

S11 Fig Footprint scores correspond to overlapping altered motif scores for several TFs. **A-B)** Bar plots of counts for footprints with corresponding motif altered by genetic variant across different DAR-classes^1^ in **A)** eWAT and **B)** liver **C-D)** Differences in footprint scores (Y-axis) across motif allelic score difference (129 vs B6) bins (X-axis) in **C)** eWAT and **D)** liver. **E-F)** Word clouds of TFs with 90% of motif altering variants presenting AC-footprints in NFR-classes^1^ of **E)** eWAT **and F)** liver. ^1^Common = ”DAR in both diet comparisons”, HFD = ”DAR in HFD comparison”, Chow = ”DAR in chow comparison”, Non-DAR = “Non-DAR in both comparisons”.
(TIF)

S12 Fig Motif altering variants are conjoint to footprint scores and CTCF binding. **A-B)** Violin plots for the number of AC-footprints per TF that were detected in DAR-classes^1^ in **A)** eWAT **and B)** liver. **C-D)** Enrichment P-values (Fisher’s test) of AC-footprints in DAR-classes^1^ for **C)** eWAT and **D)** liver, comparing Chow, HFD and Common DARs to Non-DARs, and Non-DARs to DARs of any class. Only TFs with P-value < 0.25 shown. **E)** Euler plot of CTCF ChIP-seq overlap of liver CTCF footprints. **F)** Scatter plot of footprint score differences between strain (Y-axis) and motif allelic scores (X-axis). Points coloured by motif change strength. Correlation results (Spearman’s correlation) annotated as text. Panels by CTCF ChIP-seq overlap. ^1^Common = ”DAR in both diet comparisons”, HFD = ”DAR in HFD comparison”, Chow = ”DAR in chow comparison”, Non-DAR = “Non-DAR in both comparisons”.
(TIF)

S1 Table DEGs detected in individual comparisons. A dedicated sheet for each of the tested comparisons presented in the document. Results for all tested genes are included.
(XLSX)

S2 Table Genetically altered TF binding motifs at corresponding differentially accessible footprint in liver and eWAT. Footprint score differences presented as maximum differences between the groups included in the NFR class. Common = ”DAR in both diet comparisons”, HFD = ”DAR in HFD comparison”, Chow = ”DAR in chow comparison”, Non-DAR = “Non-DAR in both comparisons”.
(XLSX)
