# Supplementary figures and images for: Genetic variation shapes the chromatin accessibility landscape and transcriptional responses in mouse adipose tissue
Source: PLoS Genet. 2026 Jan 16;22(1):e1011716. doi: 10.1371/journal.pgen.1011716 (PMC12844536; doi:10.1371/journal.pgen.1011716)

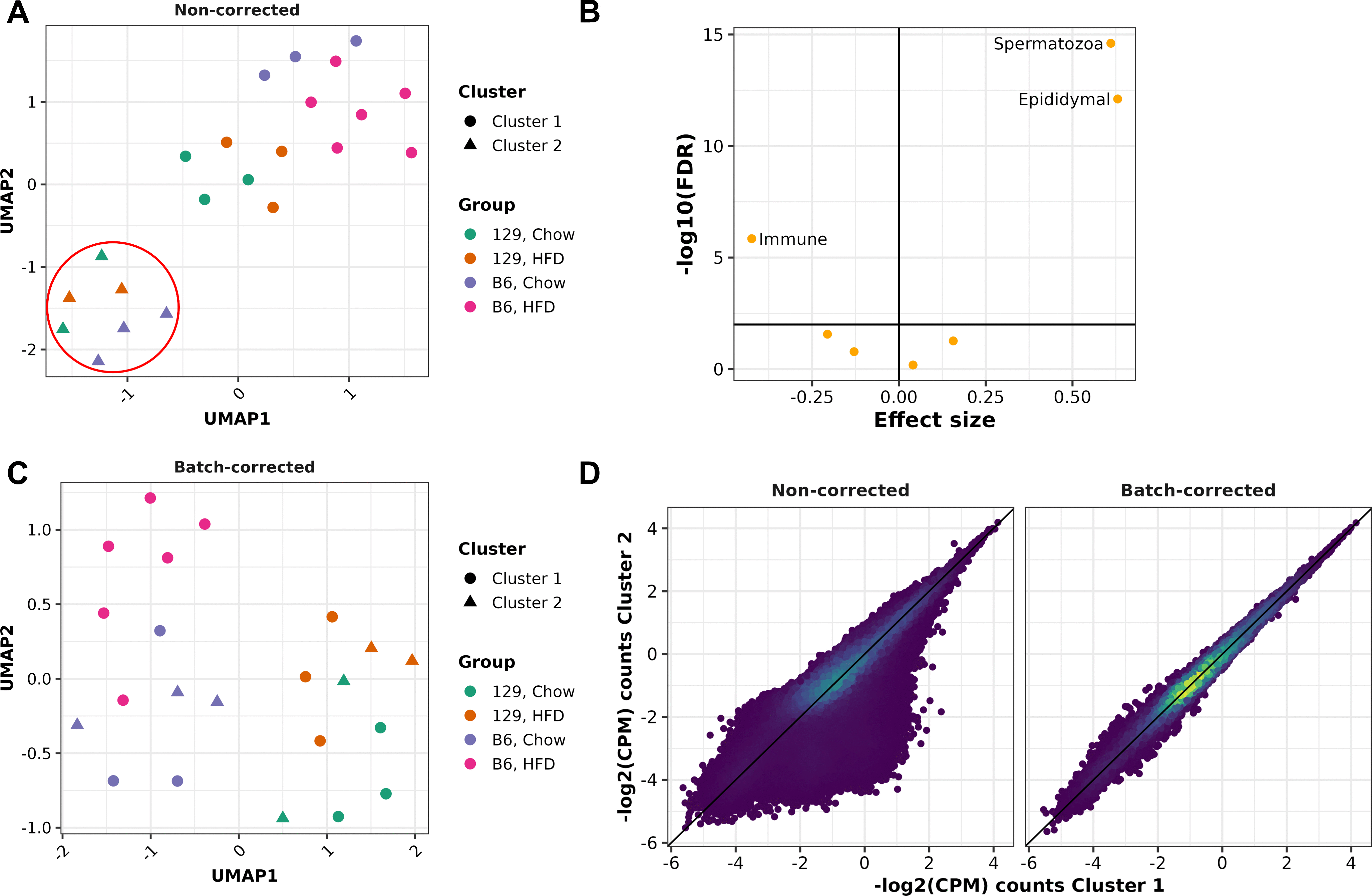

Supplement: S1 Fig — A) UMAP plot based on normalized DAR counts. Cluster 2 is highlighted with red circle. B) Chipenrich results for Cluster 2 vs Cluster 1 DARs to cell type-specific genes. Significantly enriched/depleted (FDR < 0.01) gene sets labelled. C) UMAP plot of limma batch corrected normalized counts. D) Scatter plots of mean normalized DAR counts in Cluster 1 (x-axis) and Cluster 2 (y-axis) before and after batch correction. (TIF) [file pgen.1011716.s001.tif]

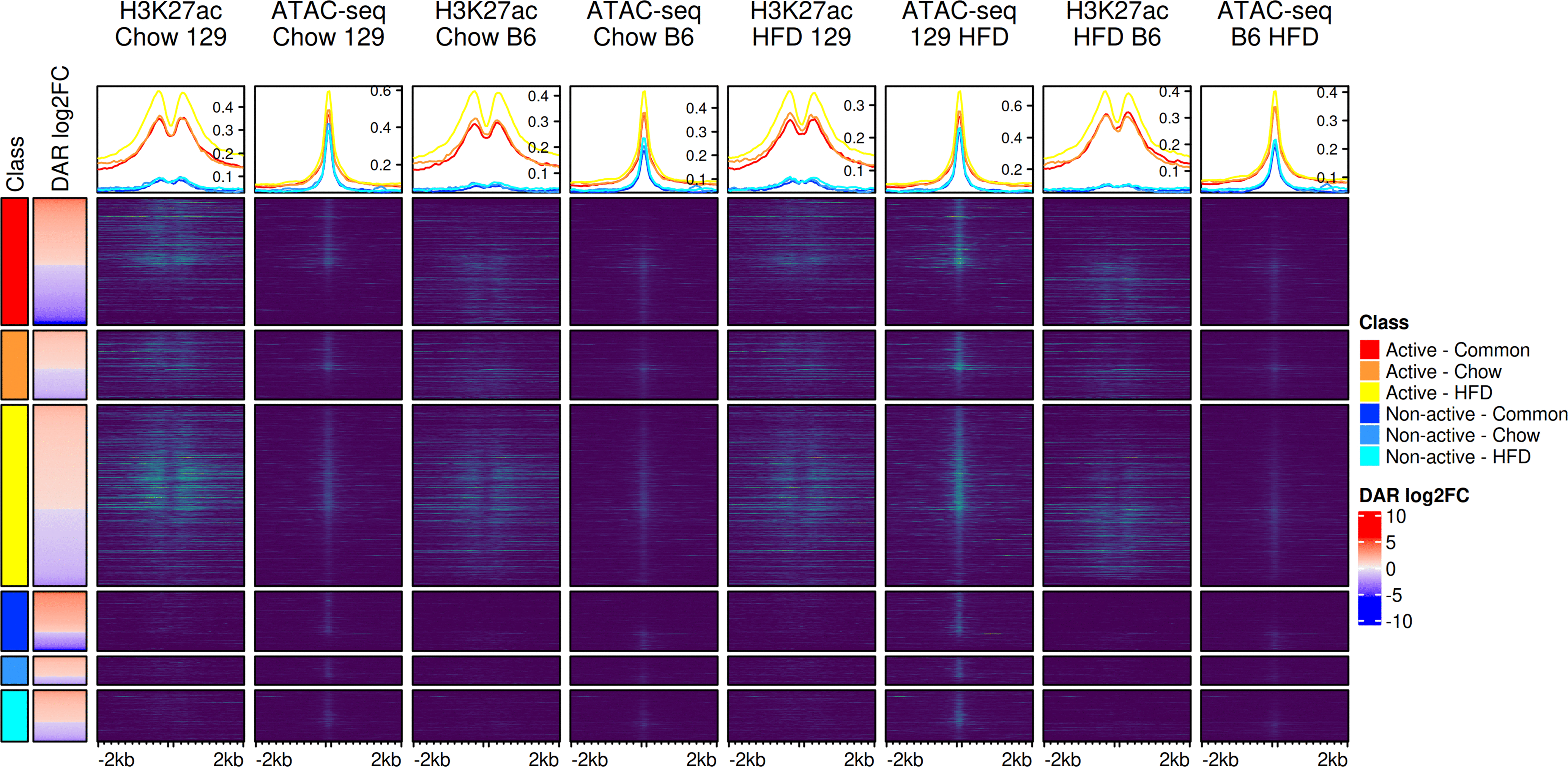

Supplement: S2 Fig — 1Common = ”DAR in both diet comparisons”, HFD = ”DAR in HFD comparison”, Chow = ”DAR in chow comparison”, Non-DAR = “Non-DAR in both comparisons”. 2Activity-class: Active = NFR flanked by H3K27ac signal, Non-active = NFR not flanked by H3K27ac signal. (TIF) [file pgen.1011716.s002.tif]

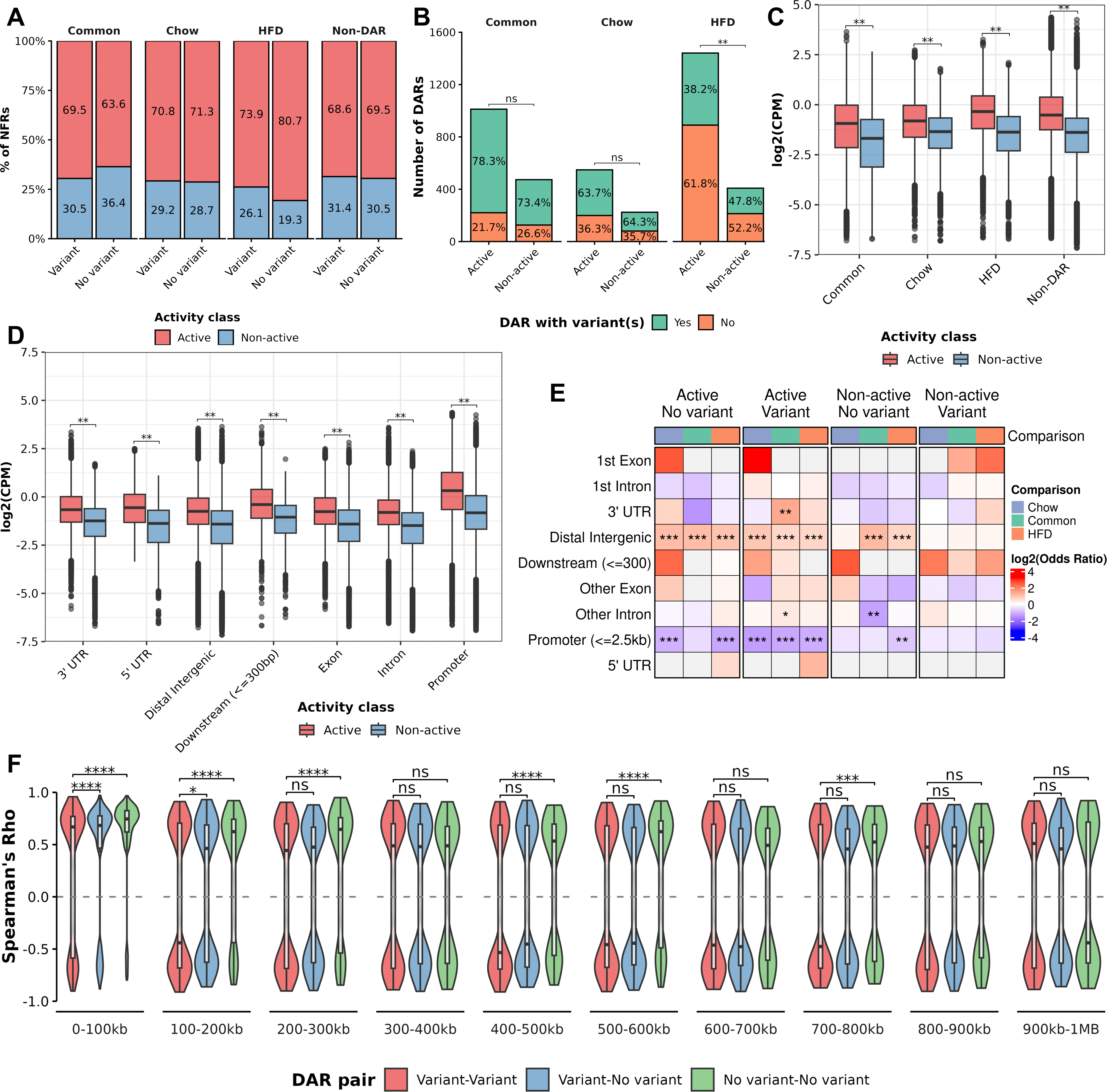

Supplement: S3 Fig — A) Fractions of NFRs in DAR-classes1 separated to activity classes2 with or without overlapping genetic variant. B) Counts of DARs in different classes1,2 with percentages of DARs overlapping with variants labelled. Statistical testing for differential proportions of variants in activity classes with Fisher’s test3. C-D) Boxplots of normalized counts for active and non-active NFRs in C) different DAR-classes1 and D) different genomic features. E) Heatmap of Fisher’s test results on the enrichment of DARs to genomic features compared to non-DARs. F) Violin plots of significant Spearman’s correlations between two DARs with or without genetic variant overlap at different distance bins. C, D, F) Statistical testing with Wilcoxon signed rank test3. 1Common = ”DAR in both diet comparisons”, HFD = ”DAR in HFD comparison”, Chow = ”DAR in chow comparison”, Non-DAR = “Non-DAR in both comparisons”. 2Activity-class: Active = NFR flanked by H3K27ac signal, Non-active = NFR not flanked by H3K27ac signal. 3P-value: * < 0.05, ** < 0.01, *** < 0.001, **** < 0.0001. (TIF) [file pgen.1011716.s003.tif]

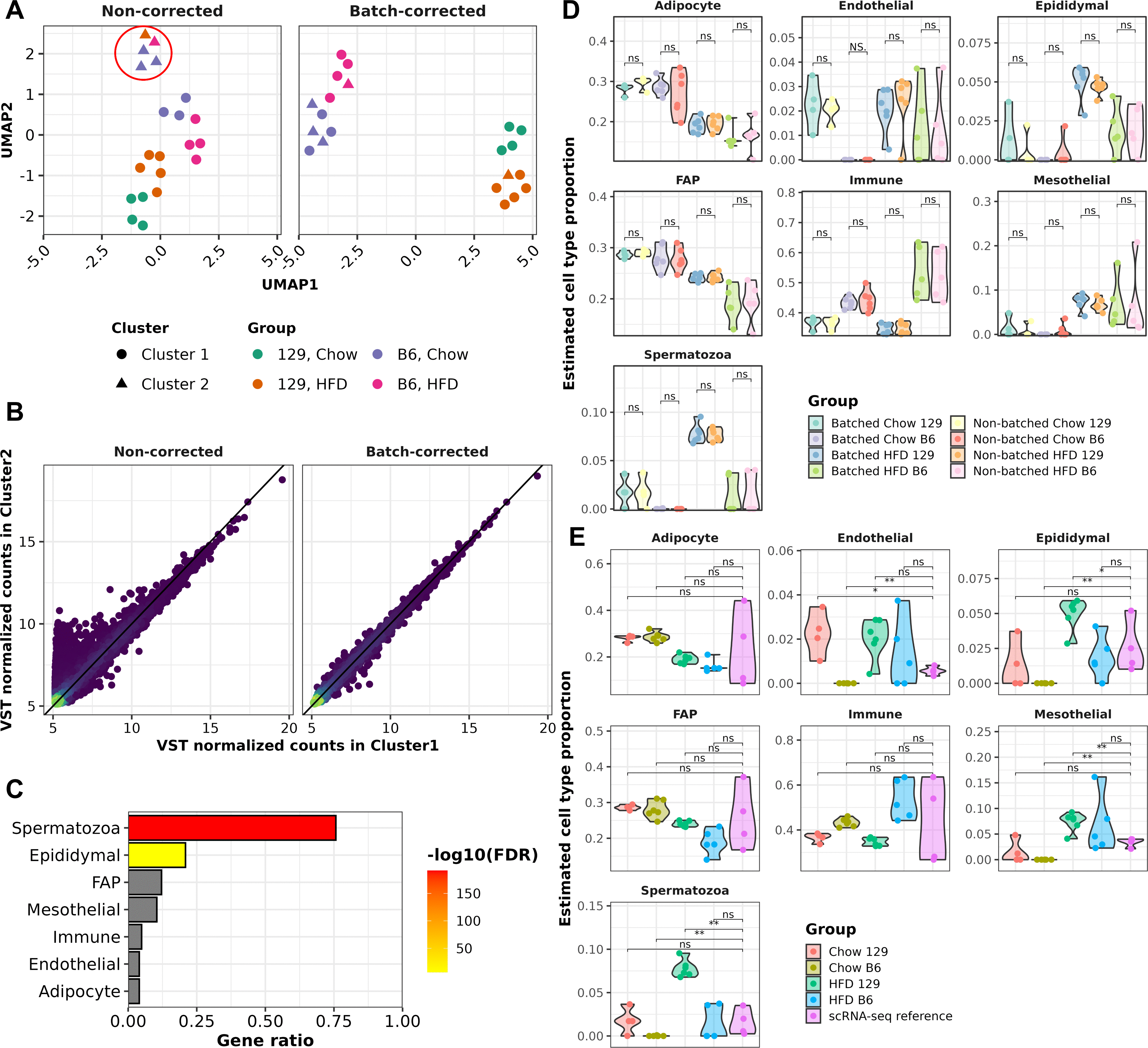

Supplement: S4 Fig — A) UMAP plot of normalized counts before and after batch correction. Cluster 2 highlighted with a red circle. B) MA plots of Cluster 2 vs Cluster 1 normalized counts before and after batch correction. C) Over representation analysis results of genes up-regulated in Cluster 2 vs Cluster 1 in sets of cell type specific genes. Bars coloured grey are non-significant (FDR > 0.01). D) Violin plots of estimated cell type proportions in batch corrected (batched) and non-corrected (non-batched) sample groups. E) Violin plots of estimated cell type proportions in batch corrected samples compared to proportions observed in the scRNA-seq reference dataset. D-E) Statistical testing with Statistical testing with Wilcoxon test. P-value: * < 0.05, ** < 0.01, *** < 0.001, **** < 0.0001. (TIF) [file pgen.1011716.s004.tif]

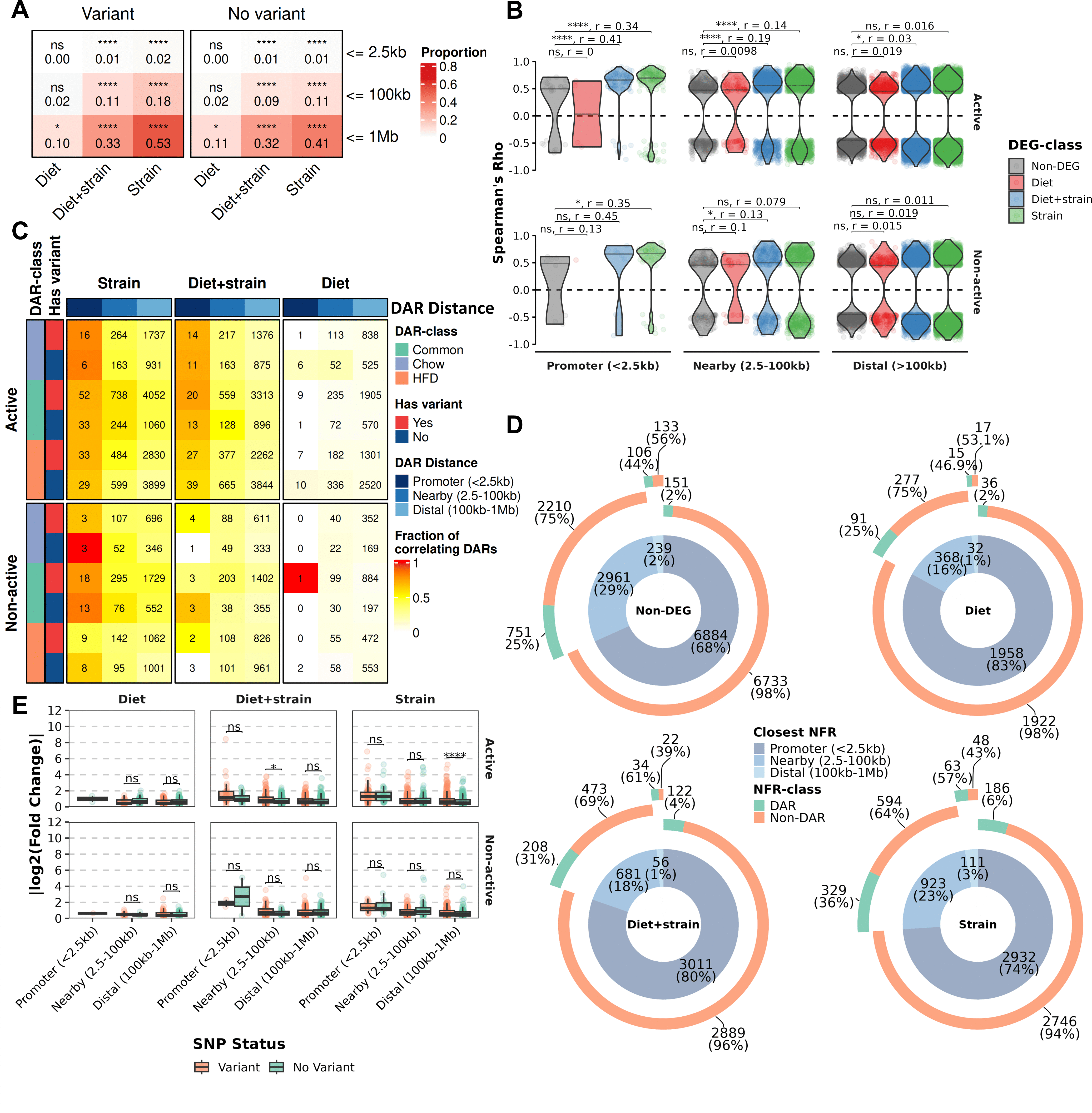

Supplement: S5 Fig — A) Heatmap displaying fractions of DEGs1 with correlating (Spearman’s Rho > 0 and P-value < 0.05) DARs (of any class) in different windows from the TSS of the DEG. Fractions and results from statistical testing (Fisher’s test2) comparing to non-DARs labelled. Panels for DARs belonging to different activity-classes3 B) Correlation coefficients (Spearman’s Rho, P-value < 0.05) for DEGs and their nearest DARs (of any class) in different distance categories. Vertical facets for DARs belonging to different activity-classes3. Statistical testing with Wilcoxon test annotated as text (r = Wilcoxon effect size) 2. C) Heatmap of fractions of correlating DARs in DAR-classes3 for DEGs in DEG-classes1 (horizontal panels) separated by Activity class4 (vertical panels), variant overlap, and distance bins. Number of linked DARs annotated as numbers in the heatmap cells. D) Circulograms for the fractions of closest NFR distance and class for expressed genes1. DAR = Chow-/HFD-/Common-DAR. E) Absolute log2 fold-changes of DEGs1 (horizontal panel) with nearest DAR (of any class) in different windows around active TSS. Separated for Activity class4 (vertical panel). Statistical testing with Wilcoxon test2. 1Non-DEG = non-DEG in all comparisons, Diet = DEG only in diet comparison, Diet+strain = DEG in both diet and strain comparisons, Strain DEGs = DEG in both strain comparisons. 2P-value: ns ≥ 0.05, * < 0.05, ** < 0.01, *** < 0.001, **** < 0.0001. 3Common = ”DAR in both diet comparisons”, HFD = ”DAR in HFD comparison”, Chow = ”DAR in chow comparison”, Non-DAR = “Non-DAR in both comparisons”. 4Activity-class: Active = NFR flanked by H3K27ac signal, Non-active = NFR not flanked by H3K27ac signal. (TIF) [file pgen.1011716.s005.tif]

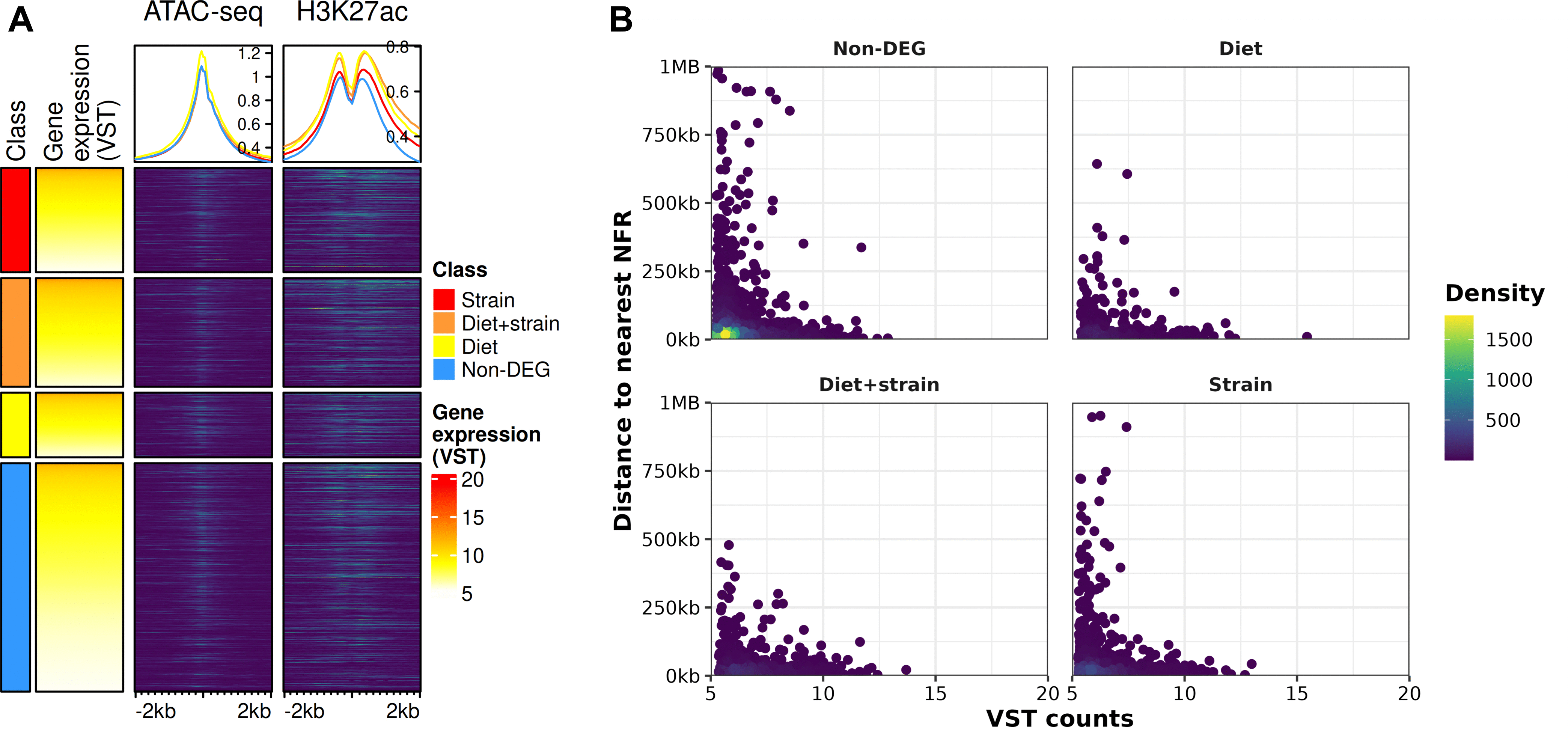

Supplement: S6 Fig — A) Enrichment heatmap of ATAC-seq and H3K27ac ChIP-seq signals ±2kb from the active TSS of expressed genes. B) Scatter plot of normalized expression levels of genes1 and their distance to nearest NFR. 1Non-DEG = non-DEG in all comparisons, Diet = DEG only in diet comparison, Diet+strain = DEG in both diet and strain comparisons, Strain DEGs = DEG in both strain comparisons. (TIF) [file pgen.1011716.s006.tif]

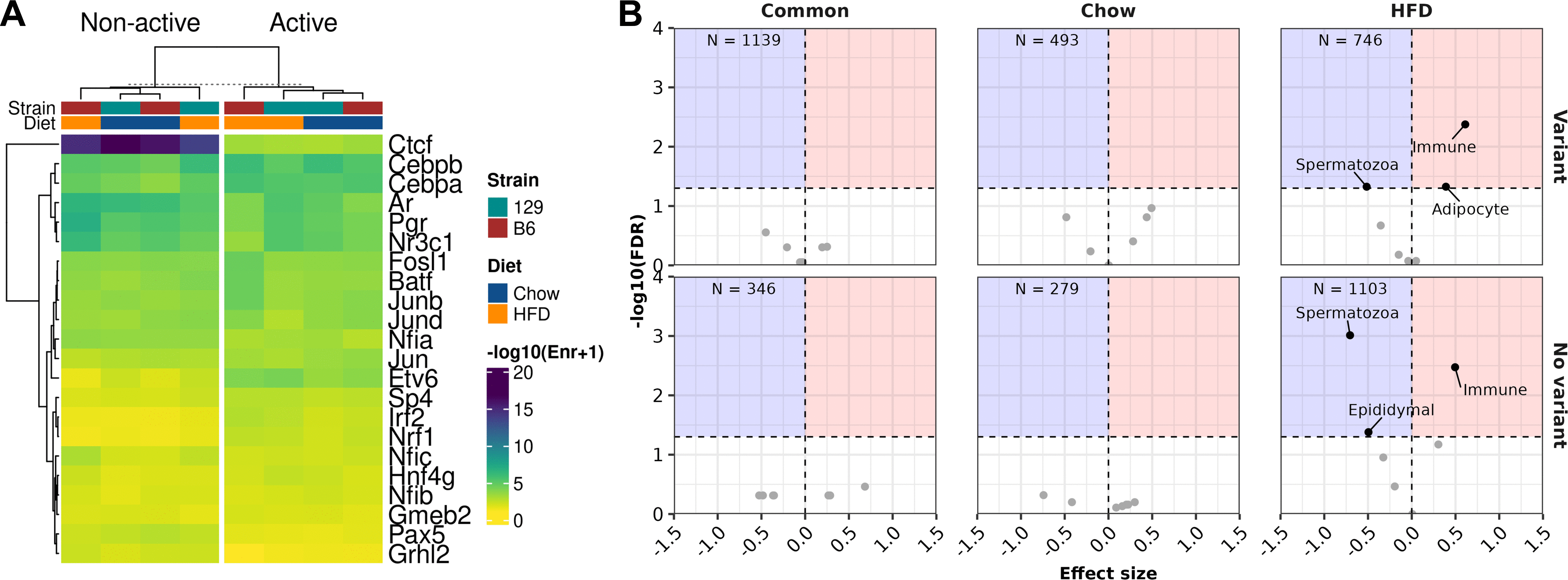

Supplement: S7 Fig — A) Motif enrichment for group-wise active and non-active NFRs. Heatmap columns and rows are clustered by Euclidean distance and columns are split using K-means clustering. Values shown are for the most significant motif for a given TF. Motifs for heatmap were selected by group-wise filtering of redundant motifs. B) Fisher’s test results for enrichment of Active and non-active DARs* to cell type specific genes. *Common = ”DAR in both diet comparisons”, HFD = ”DAR in HFD comparison”, Chow = ”DAR in chow comparison”. (TIF) [file pgen.1011716.s007.tif]

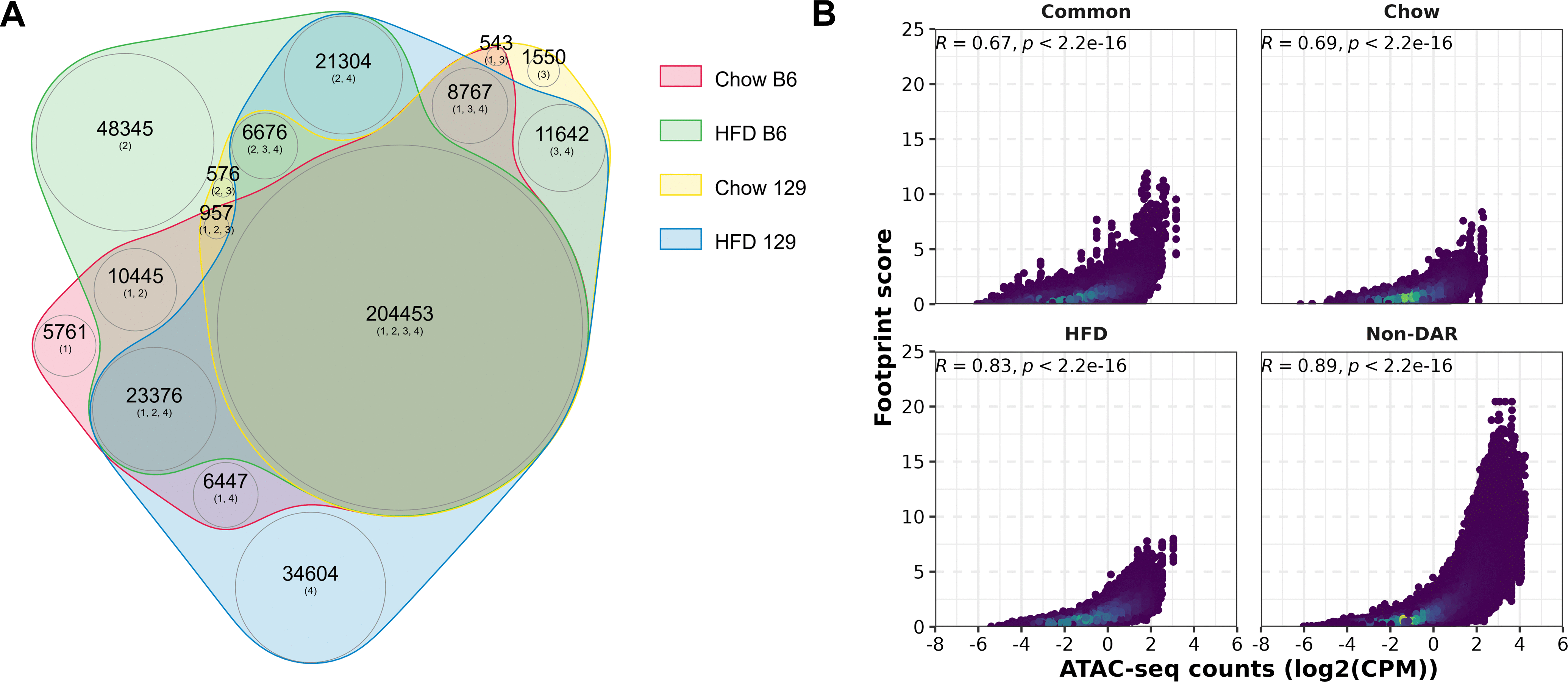

Supplement: S8 Fig — A) Venn diagram of footprints observed in the different groups in eWAT. Numeric IDs for overlapping groups are presented in the brackets. 1 = Chow B6, 2 = HFD B6, 3 = Chow 129 and 4 = HFD 129. B) Scatter plot of footprint scores and normalized (log2 CPM) ATAC-seq counts in the different NFRs1. Correlation testing with Spearman’s correlation. 1Common = ”DAR in both diet comparisons”, HFD = ”DAR in HFD comparison”, Chow = ”DAR in chow comparison”, Non-DAR = “Non-DAR in both comparisons”. (TIF) [file pgen.1011716.s008.tif]

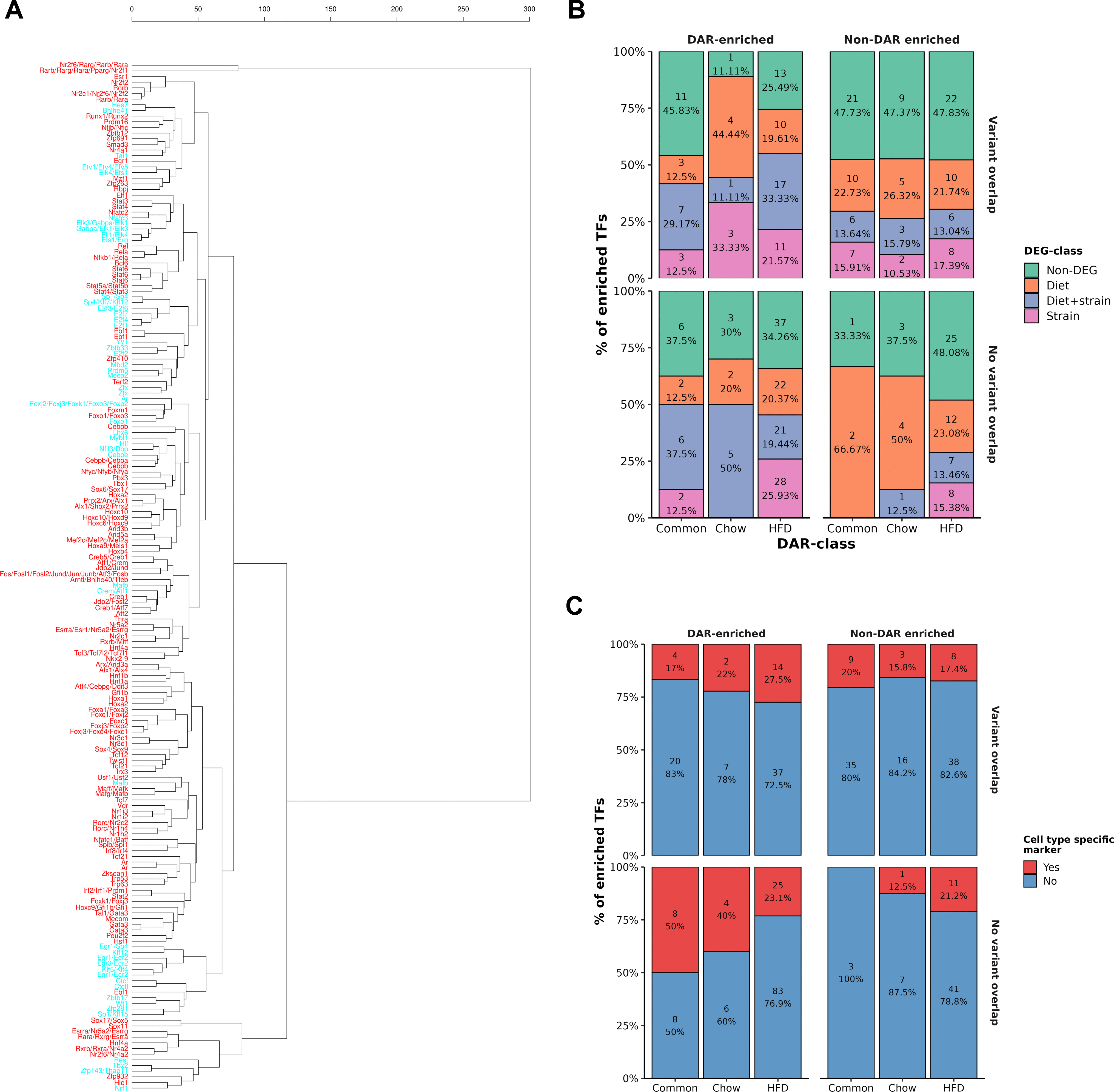

Supplement: S9 Fig — A) Motifs clustered by Pearson correlation. Motifs enriched in DARs in red and non-DARs in cyan. B) Percentages of enriched TFs in DEG-classes1. Counts and percentages labelled. C) Proportions of enriched TFs that are positive cell type markers derived from a single cell reference (Adjusted P-value < 0.01 and average log2 fold change > 0.25). Counts and percentages labelled. 1Non-DEG = non-DEG in all comparisons, Diet = DEG only in diet comparison, Diet+strain = DEG in both diet and strain comparisons, Strain DEGs = DEG in both strain comparisons. (TIF) [file pgen.1011716.s009.tif]

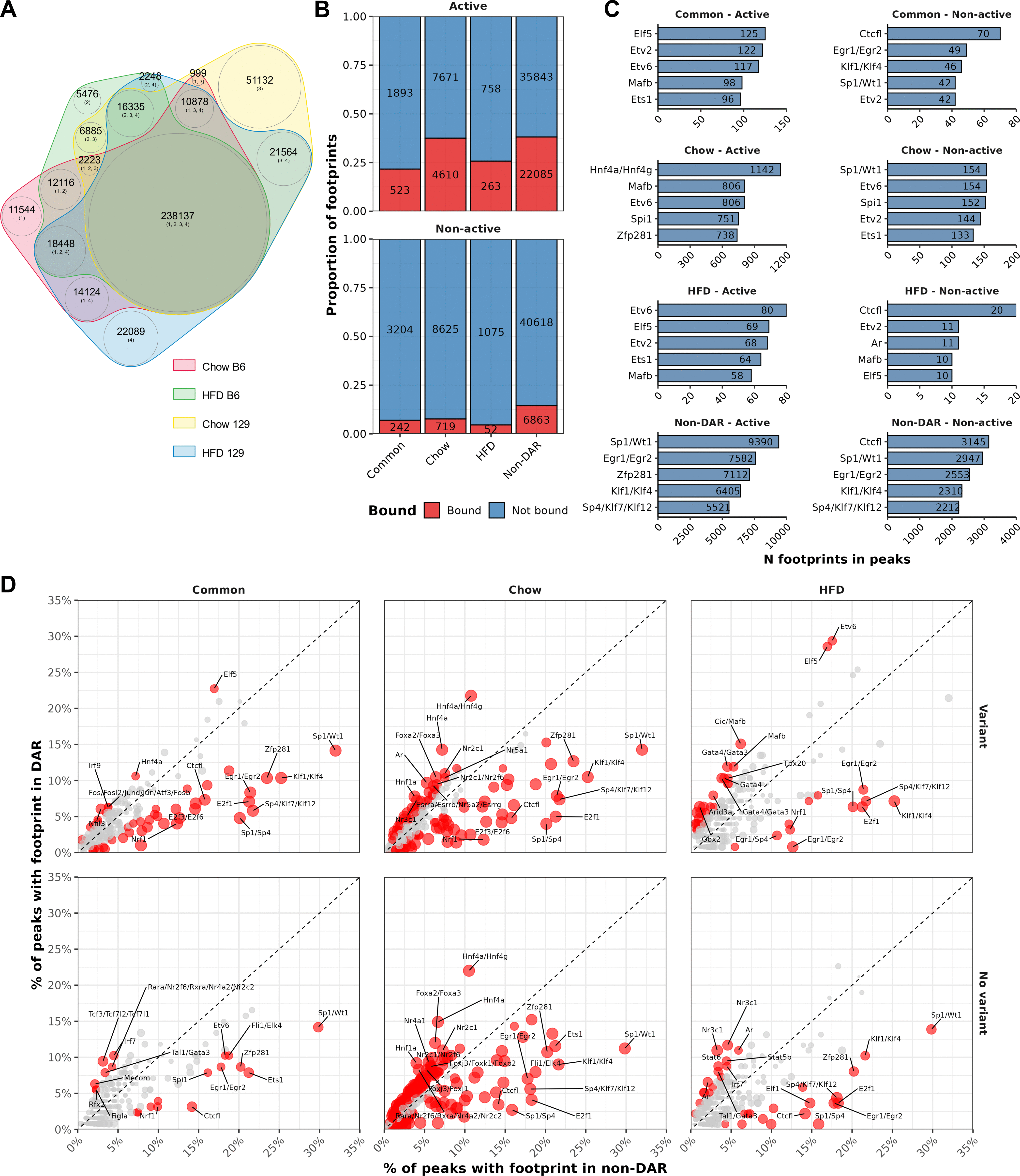

Supplement: S10 Fig — A) Venn diagram of footprints observed in the different NFRs1 in liver. Numeric IDs for overlapping groups are presented in the brackets. 1 = Chow B6, 2 = HFD B6, 3 = Chow 129 and 4 = HFD 129. B) Fractions of active and non-active NFRs by NFR-class1 that overlap with at least one footprint in liver. C) Counts for top 5 most common footprints in active and non-active NFRs1 in liver. D) Scatter plot of footprint occupancy in DARs vs non-DARs. Size of points is determined by Fisher’s test P-value; red: P-value < 0.05. Top 10 TFs with highest difference in occupancy fraction between DAR-class1 and non-DAR are labelled (P-value < 0.05). 1Common = ”DAR in both diet comparisons”, HFD = ”DAR in HFD comparison”, Chow = ”DAR in chow comparison”, Non-DAR = “Non-DAR in both comparisons”. (TIF) [file pgen.1011716.s010.tif]

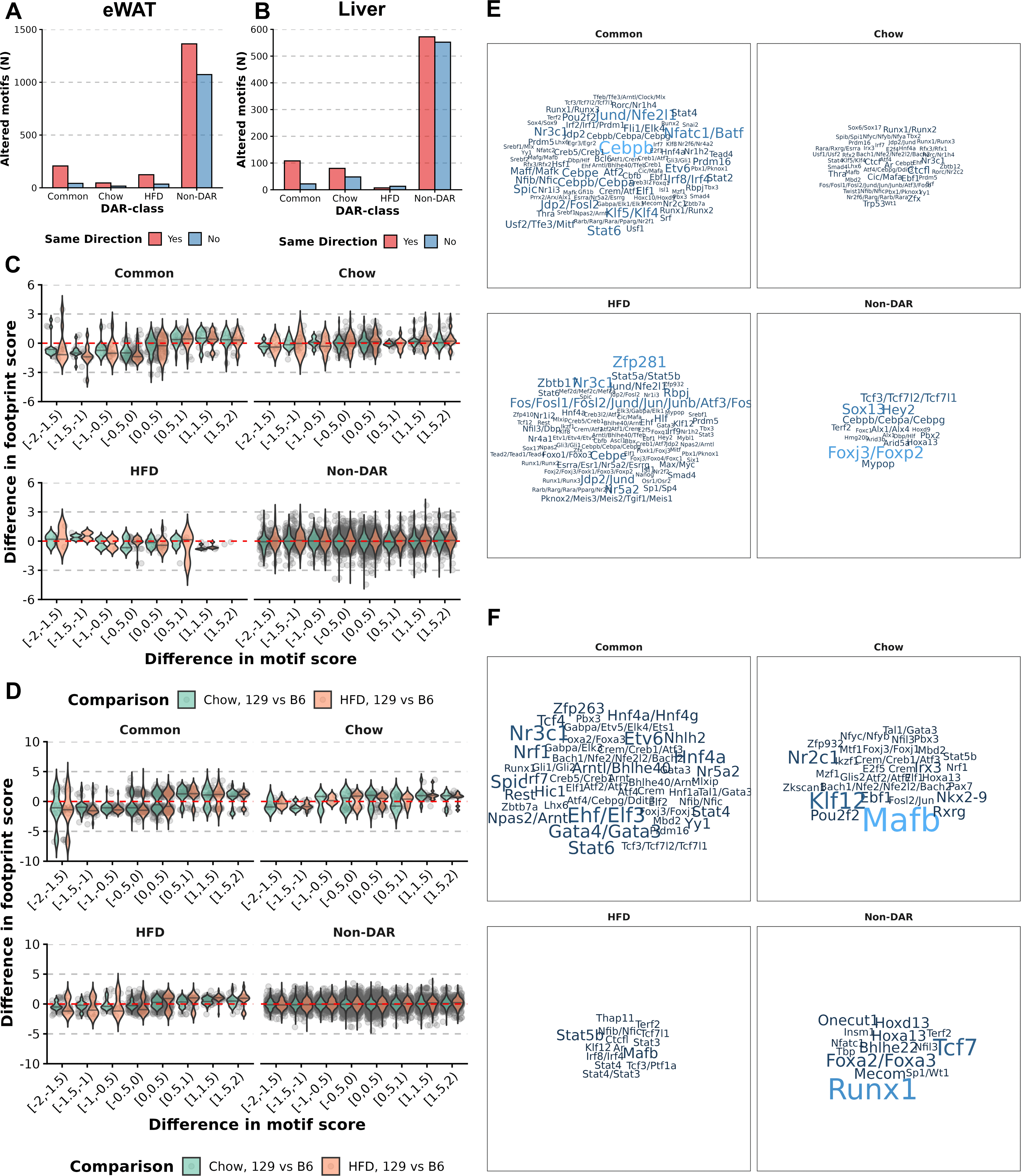

Supplement: S11 Fig — A-B) Bar plots of counts for footprints with corresponding motif altered by genetic variant across different DAR-classes1 in A) eWAT and B) liver C-D) Differences in footprint scores (Y-axis) across motif allelic score difference (129 vs B6) bins (X-axis) in C) eWAT and D) liver. E-F) Word clouds of TFs with 90% of motif altering variants presenting AC-footprints in NFR-classes1 of E) eWAT and F) liver. 1Common = ”DAR in both diet comparisons”, HFD = ”DAR in HFD comparison”, Chow = ”DAR in chow comparison”, Non-DAR = “Non-DAR in both comparisons”. (TIF) [file pgen.1011716.s011.tif]

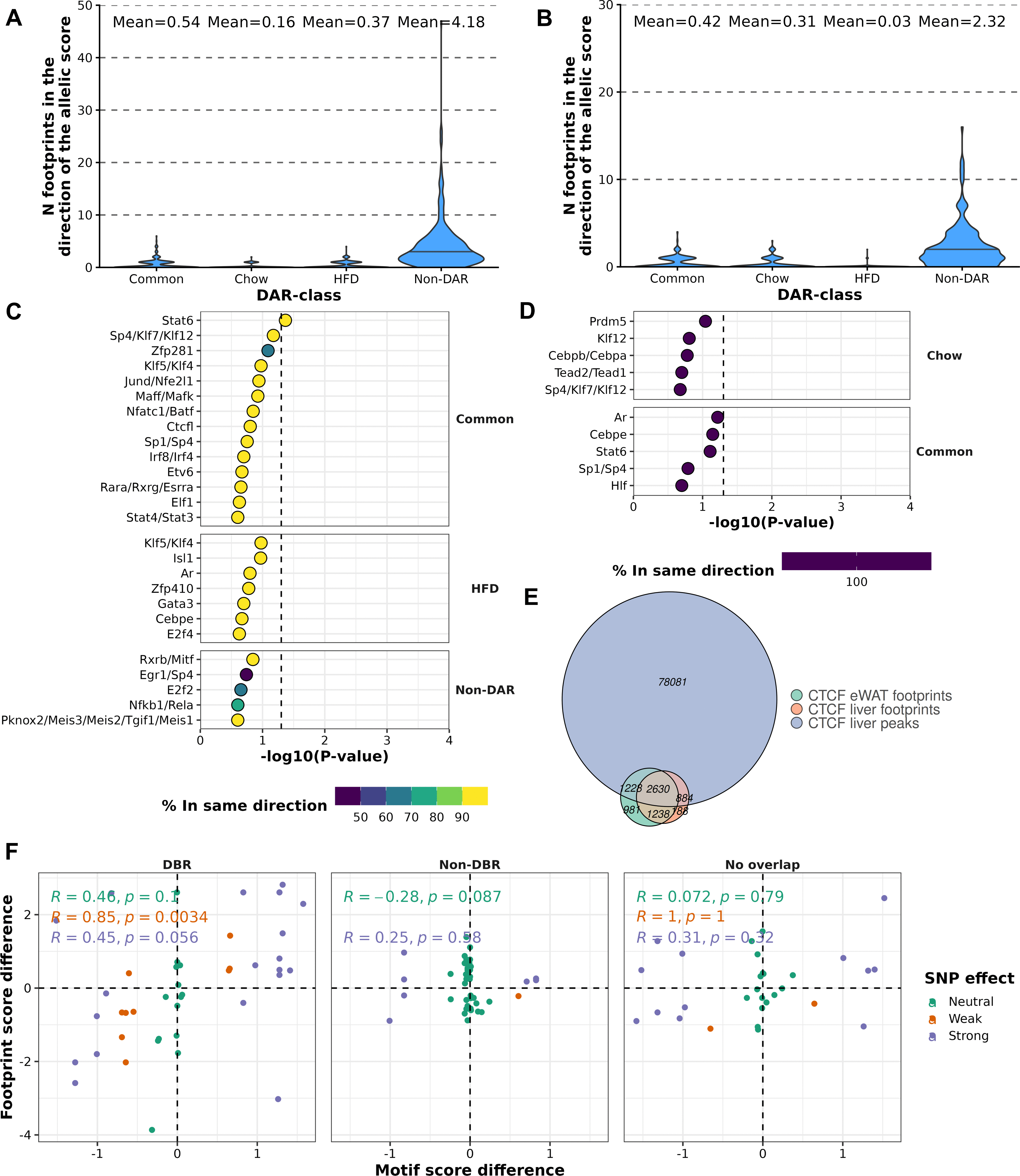

Supplement: S12 Fig — A-B) Violin plots for the number of AC-footprints per TF that were detected in DAR-classes1 in A) eWAT and B) liver. C-D) Enrichment P-values (Fisher’s test) of AC-footprints in DAR-classes1 for C) eWAT and D) liver, comparing Chow, HFD and Common DARs to Non-DARs, and Non-DARs to DARs of any class. Only TFs with P-value < 0.25 shown. E) Euler plot of CTCF ChIP-seq overlap of liver CTCF footprints. F) Scatter plot of footprint score differences between strain (Y-axis) and motif allelic scores (X-axis). Points coloured by motif change strength. Correlation results (Spearman’s correlation) annotated as text. Panels by CTCF ChIP-seq overlap. 1Common = ”DAR in both diet comparisons”, HFD = ”DAR in HFD comparison”, Chow = ”DAR in chow comparison”, Non-DAR = “Non-DAR in both comparisons”. (TIF) [file pgen.1011716.s012.tif]
